# CXCL12-mediated monocyte transmigration into brain perivascular space leads to neuroinflammation and memory deficit in neuropathic pain

**DOI:** 10.7150/thno.44364

**Published:** 2021-01-01

**Authors:** Chun-Lin Mai, Zhi Tan, Ya-Nan Xu, Jing-Jun Zhang, Zhen-Hua Huang, Dong Wang, Hui Zhang, Wen-Shan Gui, Jun Zhang, Zhen-Jia Lin, Ying-Tong Meng, Xiao Wei, Ying-Tao Jie, Peter M. Grace, Long-Jun Wu, Li-Jun Zhou, Xian-Guo Liu

**Affiliations:** 1 Department of Physiology and Pain Research Center, Zhongshan School of Medicine, Sun Yat-sen University, Guangzhou 510080, China.; 2 Guangdong Province Key Laboratory of Brain Function and Disease, Guangzhou 510080, China.; 3 Department of Anesthesiology and Pain Clinic, the First Affiliated Hospital of Sun Yat-Sen University, Guangzhou, 510080, China.; 4 Division of Emergency Medicine, the First Affiliated Hospital of Sun Yat-Sen University, Guangzhou, 510080, China.; 5 Department of Clinical Laboratory, The First Affiliated Hospital, Sun Yat-sen University, Guangzhou, 510080, China.; 6 Department of Anesthesiology, Guangdong Second Provincial General Hospital, Guangzhou, 510317, China.; 7 Department of Critical Care & Respiratory Care Research (PMG), University of Texas MD Anderson Cancer Center, Houston, Texas, USA.; 8 Department of Neurology, Mayo Clinic, Rochester, MN 55905, USA.

**Keywords:** memory deficit, chronic pain, monocyte transmigration, hippocampus perivascular macrophage, neuroinflammation, CXCL12

## Abstract

Emerging clinical and experimental evidence demonstrates that neuroinflammation plays an important role in cognitive impairment associated with neuropathic pain. However, how peripheral nerve challenge induces remote inflammation in the brain remains largely unknown.

**Methods:** The circulating leukocytes and plasma C-X-C motif chemokine 12 (CXCL12) and brain perivascular macrophages (PVMs) were analyzed by flow cytometry, Western blotting, ELISA, and immunostaining in spared nerve injury (SNI) mice. The memory function was evaluated with a novel object recognition test (NORT) in mice and with Montreal Cognitive Assessment (MoCA) in chronic pain patients.

**Results:** The classical monocytes and CXCL12 in the blood, PVMs in the perivascular space, and gliosis in the brain, particularly in the hippocampus, were persistently increased following SNI in mice. Using the transgenic CCR2^RFP/+^ and CX3CR1^GFP/+^ mice, we discovered that at least some of the PVMs were recruited from circulating monocytes. The SNI-induced increase in hippocampal PVMs, gliosis, and memory decline were substantially prevented by either depleting circulating monocytes via intravenous injection of clodronate liposomes or blockade of CXCL12-CXCR4 signaling. On the contrary, intravenous injection of CXCL12 at a pathological concentration in naïve mice mimicked SNI effects. Significantly, we found that circulating monocytes and plasma CXCL12 were elevated in chronic pain patients, and both of them were closely correlated with memory decline.

**Conclusion:** CXCL12-mediated monocyte recruitment into the perivascular space is critical for neuroinflammation and the resultant cognitive impairment in neuropathic pain.

## Introduction

A compelling body of clinical and experimental evidence indicates that chronic pain is often comorbid with cognitive impairment 1-5. The cognitive impairment is associated with high-impact chronic pain, a disabled subgroup of chronic pain patients 6. However, the mechanisms underlying the comorbidity remain contested. It has been proposed that pain-related sensory inputs may decline memory by disrupting attention, important for working memory formation 7, 8. Yet, this hypothesis is challenged by the evidence that acute pain does not produce cognitive deficit 9, and that relief of pain with analgesics in chronic patients failed to improve their cognitive function 10. We and others have provided evidence suggesting that structural and functional impairments of the hippocampus underlie cognitive deficit associated with chronic pain. Chronic neuropathic pain induced by spared nerve injury (SNI) inhibits long-term potentiation 1 and reduces the number of excitatory synapses 11 in the hippocampus of rodents. Consistently, the hippocampal volume is substantially reduced in human patients with chronic back pain and complex regional pain syndrome compared to healthy individuals 3.

Multiple animal models of cognitive impairment are characterized by sustained activation of microglia and/or astrocytes and overproduction of proinflammatory cytokines, impairing neurogenesis and synaptic plasticity 5, 12-15. We and others have shown that SNI causes neuroinflammation in several brain regions, including the hippocampus 1, 16-18. Genetic deletion of microglia, tumor necrosis factor (TNF-α) receptor 1, or interleukin-1beta (IL-1β) receptor 1 prevents hippocampus dysfunction of and memory deficit induced by SNI. The data suggested that neuroinflammation is a root cause of SNI-induced hippocampus dysfunction and cognitive deficit. However, how peripheral nerve injury remotely induces neuroinflammation in the brain, particularly in the hippocampus, remains poorly understood.

Based on the findings from other cognitive deficit models 19-21, we hypothesized that the infiltration of circulating monocytes into the hippocampus might be essential for inducing neuroinflammation and memory decline in neuropathic conditions. We found that the classical monocytes in blood and perivascular macrophages (PVMs) in many brain regions, particularly around hippocampal sulcus, were persistently increased following SNI. The transformation of circulating monocytes into PVMs mediated by CXCL12 was crucial for neuroinflammation and memory impairment in neuropathic pain.

## Materials and Methods

### Animals

Adult C57BL/6J mice (7-10 weeks old) were obtained from the Institute of Experimental Animals of Sun Yat-sen University (China). Adult CCR2^RFP/+^:CX3CR1^GFP/+^ animals were generated by crossing CX3CR1^GFP/GFP^/CCR2^RFP/RFP^ with C57BL/6J mice (obtained from Dr. Long-Jun Wu, Mayo Clinic, Minnesota, USA). The animals were housed in a 12 h light/dark cycle (lights on 07:00) with access to food and water* ad libitum*. The room temperature was maintained at 23 ± 2 °C and humidity at 50~60%. The experiments were performed mainly with adult male mice. Both male and female mice were used for sex difference analysis, as indicated. The animals were randomly assigned to different treatment groups. All experimental procedures were approved by the Institutional Animal Care and Use Committee (IACUC) at Sun Yat-Sen University (China) and Mayo Clinic (USA).

### Spared nerve injury (SNI)

SNI surgery was performed as described in previous studies 11, 22. Anesthesia was induced and maintained with 2% and 1.0% isoflurane in 100% oxygen, respectively, administrated with R405 Mouse Ventilator (RWD Life Science, China) in a transparent acrylic chamber. SNI surgeries were completed within 20 min and may not affect cognitive function in mice 23. An incision on the lateral surface of the thigh was made, and the left sciatic nerve and its three terminal branches were separated. The common peroneal and the tibial nerves were ligated with 5-0 suture and transected distal to the ligation. The touch or pull of the sural nerve was carefully avoided. The sham operation was performed by exposing the three terminal branches of the sciatic nerve without any nerve damage.

### Flow Cytometry

#### Collection of plasma and leukocytes

The blood was collected at 10:00~12:00 AM. Under urethane (1.7 g/kg, i.p.) anesthesia, thoracotomy was performed, and the whole blood was collected by cardiac puncture into EDTA tubes. Subsequently, blood samples were centrifuged at 400 ×g for 20 minutes at 4 °C to separate plasma and blood cells. Plasma samples were stored at -80 °C for further analysis and the blood cells were diluted with PBS. The red blood cells (RBC) were lysed using the RBC Lysis Buffer (420301, Biolegend, USA) and leukocytes were used for flow cytometry.

### Flow cytometry for leukocytes

Leukocytes were washed and the cell surface antigens were stained with corresponding antibodies, as previously described 24. In brief, leukocytes were blocked with 2% goat serum and then incubated with anti-CD45, anti-CD11b, anti-Ly6C, anti-Ly6G, anti-CD3, and anti-CD49b antibodies (Table S1). Data acquisition was performed on a flow cytometer (CytoFLEX S, Beckman Coulter, USA) and analyzed with CytExpert software. According to the expression of surface antigens, the ratios of all subtypes of blood leukocytes were determined by gating from CD45^+^ cells (see Figure S1).

### Immunofluorescence

Animals were deeply anesthetized with urethane (1.7 g/kg, i.p.) and perfused transcardially with PBS until the outflow from right auricle became clear, and then with cold 4% paraformaldehyde solution. After the perfusion, the brain tissues were removed and fixed with 4% paraformaldehyde solution for 3 h at 4 °C and 30% sucrose for 72 h. The fixed brain tissues were cut with a cryostat microtome (Leica CM3050 S, Germany). Cryostat sections (25 μm in thickness) were blocked with 5% goat serum in 0.3% Triton X-100 (Cat# T9284, Sigma, Sigma-Aldrich, Germany) TBS buffer for 1 h. Subsequently, the sections were incubated with primary antibodies overnight at 4 °C and the secondary antibody for 1 h at room temperature. Single- or double-immunofluorescence staining was performed by using anti-CD68, anti-CXCL12, anti-PECAM-1, anti-GFAP, anti-CD11b, anti-P2Y12, anti-CD13, anti-CD3, anti-CD49b, and anti-Ly6G antibodies. Secondary antibodies conjugated to Alexa-488, 555, or 647 were used at 1:500 dilutions. The details of the antibodies used in this study are presented in Table S1. The coverslips were mounted with Fluoromount-G with Dapi (Cat# 0100-20, Southern Biotech, USA). The fluorescent images were obtained with EVOS (EVOS FL Imaging System, Thermo Fisher Scientific, USA) microscope and confocal microscope (Nikon C2, Japan).

### Quantification of immunofluorescence staining

Fluorescence signal intensity was quantified by investigators blinded to treatment conditions using Image J software (National Institutes of Health, Bethesda, MD). For an unbiased representation of the images, all laser power parameters, pinhole size and image detection were kept constant for all samples. The fluorescence integrated intensity (IntDen) in 10× and 63× images was respectively calculated. To analyze the difference in fluorescent IntDen, a fixed intensity threshold was set appropriately and kept constant in all groups. Fluorescent IntDen of PECAM-1, CD11b, GFAP, and perivascular CXCL12 CD68^high^ or CA1 CXCL12 proteins from the sham or vehicle group were set as 1 baseline. The data from other groups were normalized with the baseline. Figure 4E shows that the raw fluorescence integrated intensity (RawIntDen) value of CD11b or GFAP was directly used to compare the signal expression among different brain regions.

CD68 was expressed in PVMs and microglia in the brain with the expression level being higher in PVMs than microglia (Figure 2B). To detect CD68^high^ signals, the threshold for integrated density was adjusted to filter out CD68^low^ signals (see Figure S2). The fluorescence CD68^high^ intensity associated uniquely with PVMs was calculated.

For the identification and quantification, the expression of CXCL12 in CA1 and lacunar regions were analyzed separately. The perivascular CXCL12 was analyzed by drawing a polygon to select the desired area, and only the vascular-like signal from lacunosum molecular layer of the hippocampus was selected, while neuron-like signals expressed in the CA1 pyramidal cell layer and granular cell layer were excluded. For CXCL12 expression analysis in CA1, a different arc polygon was drawn to select the CA1 area.

The number of microglia marked by CD11b, astrocytes by GFAP, or CD68^high^ cells in immunofluorescence photographs were determined by counting positive cells with a clear nuclear profile using the Image J image processing and analysis program. To quantify immunoreactivity profiles, 3-4 sections per mouse from 3 mice were selected randomly for each group in a blind fashion.

### Evaluation of the blood-brain barrier (BBB) permeability

We used Evans blue (EB, 961 Da that easily binds to albumin, ~69 KDa) as a macromolecular tracer and fluorescein sodium (NaFlu, 376 Da) as a micromolecular tracer to evaluate the BBB permeability, as previously described 25. EB (2% w/v in saline, 4 mL/kg; Cat# E2129-10G, Sigma-Aldrich, Germany) or NaFlu (5%, 4 mL/kg; Cat# F6377, Sigma-Aldrich) was injected through the tail-vein under anesthesia with urethane (1.7 g/kg, i.p.). Forty-five minutes after injection, mice were thoroughly perfused with PBS and the hippocampi were immediately dissected. EB samples were extracted by incubating the tissue in 500 μL of formamide at 60 °C for 72 h. The EB concentration was measured by spectrophotometry at 620 nm. For the measurement of NaFlu in hippocampi, the tissues were homogenized in 0.25 mL of PBS. An equal volume of 60% trichloroacetic acid was added to the tissue homogenate and mixed with a vortex for 2 min to precipitate proteins. After 30 min at 4 °C, samples were centrifuged for 10 min (14000 × g, 4 °C). NaFlu concentration in the supernatant was measured by spectrophotofluorimetry at the excitation wavelength of 440 nm and emission wavelength of 525 nm.

### Western blotting

Under deep anesthesia with urethane, animals were perfused transcardially with cold PBS, and brain tissues were dissected, homogenized, and sonicated in lysis buffer with protease inhibitor cocktail (Cat# 05892970001, Roche Molecular Biochemicals, Germany). Total proteins (30 μg) were loaded per well, separated by SDS-PAGE, and transferred to a PDVF membrane. The blots were blocked with 5% bovine serum albumin in TBST (Tris-buffered saline, 0.1% Tween 20) at room temperature for 1 h, and then incubated overnight at 4 °C with primary anti-ZO-1, anti-occludin, anti-CXCL12, and anti-β-actin antibodies (Table S1). Subsequently, the blots incubated with horseradish peroxidase-conjugated IgG at room temperature for 1 h. The proteins were detected by chemiluminescence (Millipore, USA) and analyzed by the Tanon-5200 Chemiluminescent Imaging System (Tanon Science and Technology, China). The integrated optical density of each immunoreactive band was measured with software Image J and then compared to β-actin. The ratio of protein/β-actin from the sham group was set as a baseline.

### RT-PCR

Bilateral hippocampi were excised from each deep urethane-anesthetized mouse and homogenized in TriZol. RNA was isolated using TriZol/chloroform extraction and cDNA was prepared from total RNA by reverse transcription with PrimeScript RT Master Mix (RR036A, Takara). Quantitative reverse transcriptase PCR (qRT-PCR) was performed with CFX 96 touch3 (Bio-rad) using TB Green premix Ex Taq (RR820A, Takara). The conditions for fast qRT-PCR were as follows: 1 cycle of 95 °C for 30s, 40 cycles of 95 °C for 5 s, and 60 °C for 30 s. At the end of the PCR, the samples were subjected to melting analysis to confirm amplicon specificity. Primer sequences were as follow: Occludin forward (5'-CCAGGCAGCGTGTTCCT-3'), Occludin reverse (5'-TTCTAAATAACAGTCACCTGAGGGC-3'); ZO-1 forward (5'-TGTGAGTCCTTCAGCTGTGGAA-3'), ZO-1 reverse (5'-GGA ACTCAACACACCACCATT G-3'); CXCL12 forward (5'-GCTCCCTTGGTTCAGAAAATTG-3'), CXCL12 reverse (5'-TCACCAGACAGGTGCCATCA-3'); β-actin forward (5'-CCACACCCGCCACCAGTTCG-3'), β-actin reverse (5'-TACAGCCCGGGGAGCATCGT-3'). The relative signal mRNA expression was normalized to β-actin. The ratio of signal mRNA/β-actin from the sham group was set as 1 and the data from other groups were analyzed using the sham baseline.

### Monocyte depletion

Clodronate liposomes (CLL, 15 mL/kg; Cat# C-010, LIPOSOMA, Clodronate Liposomes.com, Amsterdam, the Netherlands) were injected into the tail vein at 1 d before and 3 d after sham or SNI surgery to deplete circulating monocytes 24. Control mice received PBS at the same time points. The efficiency of clodronate to deplete monocyte/macrophages in normal mice was evaluated by blood flow cytometry and hippocampal immunofluorescence staining.

### Novel object recognition test (NORT)

Our previous study showed that SNI impairs both working memory and short-term memory (STM) but does not affect reference memory and long-term memory 1. In this study, we used NORT to analyze STM in mice. Briefly, the apparatus consisted of a round arena (diameter: 50 cm) with white walls and floor. The box and objects were cleaned between trials to prevent the build-up of olfactory cues. Animals received two sessions of 10 min each in the empty box to habituate them to the apparatus and test room. After 24 h, each animal was placed in the white box and exposed to two exact same objects for 5 minutes at first. After a retention interval for 10 minutes, the less preferential object was replaced by a novel object, and 5 minutes were allowed for each animal to explore the two different objects. The recognition index was calculated as the ratio of time spent exploring the novel object over total exploration time. Experimenters were blind to treatments.

### Plasma cytokine measurement

Cytokines were measured using commercially available mouse cytokine array kits from R&D Systems (Cat# ARY006, MN, USA) and CXCL12 was analyzed with the mouse CXCL12 ELISA kit (Cat# ab100741, Abcam, Cambridge, UK) and human CXCL12 ELISA assay (Cat# CHE0068, 4A biotech, Beijing, China). Briefly, 100 μL of each plasma sample was applied to the mouse cytokine array while 50 μL mice/75 μL human plasma sample was used to estimate the concentrations of CXCL12 in the ELISA experiment according to the manufacturer's specification.

### Injection of CXCL12 neutralizing antibody, AMD3100 and CXCL12 protein

CXCL12 neutralizing antibody (Cat# ab9797, Abcam, Cambridge, UK) was intravenously (i.v.) injected. To yield one-half maximal inhibition of the biological activity of CXCL12, the concentration of the antibody was 20-40 times higher than that of CXCL12, as per the manufacturer's instructions. Since the plasma concentration of CXCL12 in SNI mice was around 200 pg/mL (Figure 6D), the antibody at a higher dose (20 ng in 200 μL saline) was i.v. injected 30 min before and daily after sham or SNI surgery for 9 consecutive days. Control mice were injected with the same volume of sterile saline (vehicle, Figure 7A).

CXCR4 antagonist AMD3100 (Cat# A5602, Sigma-Aldrich, Darmstadt, Germany) was dissolved with sterile saline at a concentration of 200 μg/ mL and i.p. injected at 1 mg/kg 30 min before and daily after SNI or sham operation for 9 consecutive days. It has previously been reported daily injection of the compound at this dose was sufficient for blocking CXCR4 without causing stem cell mobilization 26.

Recombinant mice CXCL12 protein (Cat# P4371, Abnova, Taipei, China) was stored at -80 °C at a concentration of 100 μg/mL and diluted in 0.1% BSA in saline to working concentrations before administration. The plasma concentration of CXCL12 was ~ 200 pg/ mL in SNI mice and ~ 60 pg/mL in naïve mice. Since the plasma volume of adult mice is ~1.0 mL, 140 pg CXCL12 should be injected to naïve mice to achieve the pathological concentration of CXCL12 observed in SNI mice. Considering the half-time of CXCL12 is less than 30 min 27, CXCL12 at 1 ng/ mL or 2.5 ng/mL in 0.2 mL was injected. The same sterile saline volume containing 0.1% BSA served as the control group (vehicle, Figure 8A).

### Montreal Cognitive Assessment scores, routine blood test, and plasma CXCl12 in human subjects

Montreal Cognitive Assessment (MoCA), which measures general cognition and is sensitive to dementia, was used to evaluate cognitive function in healthy controls and chronic pain patients. A previous report showed that the MoCA score was positively correlated with a larger hippocampal volume among healthy older adults 28. A total of 30 chronic neuropathic pain patients with chronic low back pain, neuralgia/r radiculopathy, osteoarthritis, or complex regional pain syndrome and 40 healthy controls were enrolled in the study (Table 1 and Table S2). All were right-handed and gave full informed consent to the experimental procedures approved by the Clinical Research Ethics Committee of Sun Yet-sen University (China) and written informed consent was obtained from all the subjects. Healthy control subjects were recruited from the general public. The patients were recruited from the pain clinic of the First Affiliated Hospital of Sun Yat-sen University (China). All chronic pain patients had a definitive diagnosis according to the International Classification of Diseases-11 29 by a specialist pain physician. The inclusion criteria included pain duration of > 3 months and intensity ≥ 4 assessed with Numeric Rating Scale (NRS). The exclusion criteria included other chronic painful conditions, history of head injury or tumor, psychiatric diseases, systemic disease, recent infection, recent extracorporeal circulation, bone marrow transplant, immunosuppressive therapy, or blood disorders. The blood samples were collected between 10:00-12:00 AM from patients and healthy individuals. The routine blood test results of all participants were analyzed with Sysmex XN-9100™ Automated Hematology System (Sysmex Corporation, Kobe, Japan) and plasma CXCL12 was measured with an ELISA assay kit.

### Statistical Analysis

Data are presented as mean ± standard error of the mean (SEM). Statistical tests were calculated using GraphPad Prism 7.0 software (GraphPad Software, CA, USA). Data were analyzed by unpaired Student's t-test (2 tails). Other values of each experimental group were tested using one-way ANOVA or two-way ANOVA, followed by Bonferroni's post-hoc comparisons (*P* < 0.05 was considered statistically significant). The Pearson correlation coefficients were calculated to determine the relationship between MoCA score and the percent of blood monocytes in total white blood cells (WBC) or plasma CXCL12 concentration.

### Data availability

The data from this study will be available on request from the corresponding author.

## Results

### SNI induces a persistent increase in circulating classical monocytes

**Figure 1:**
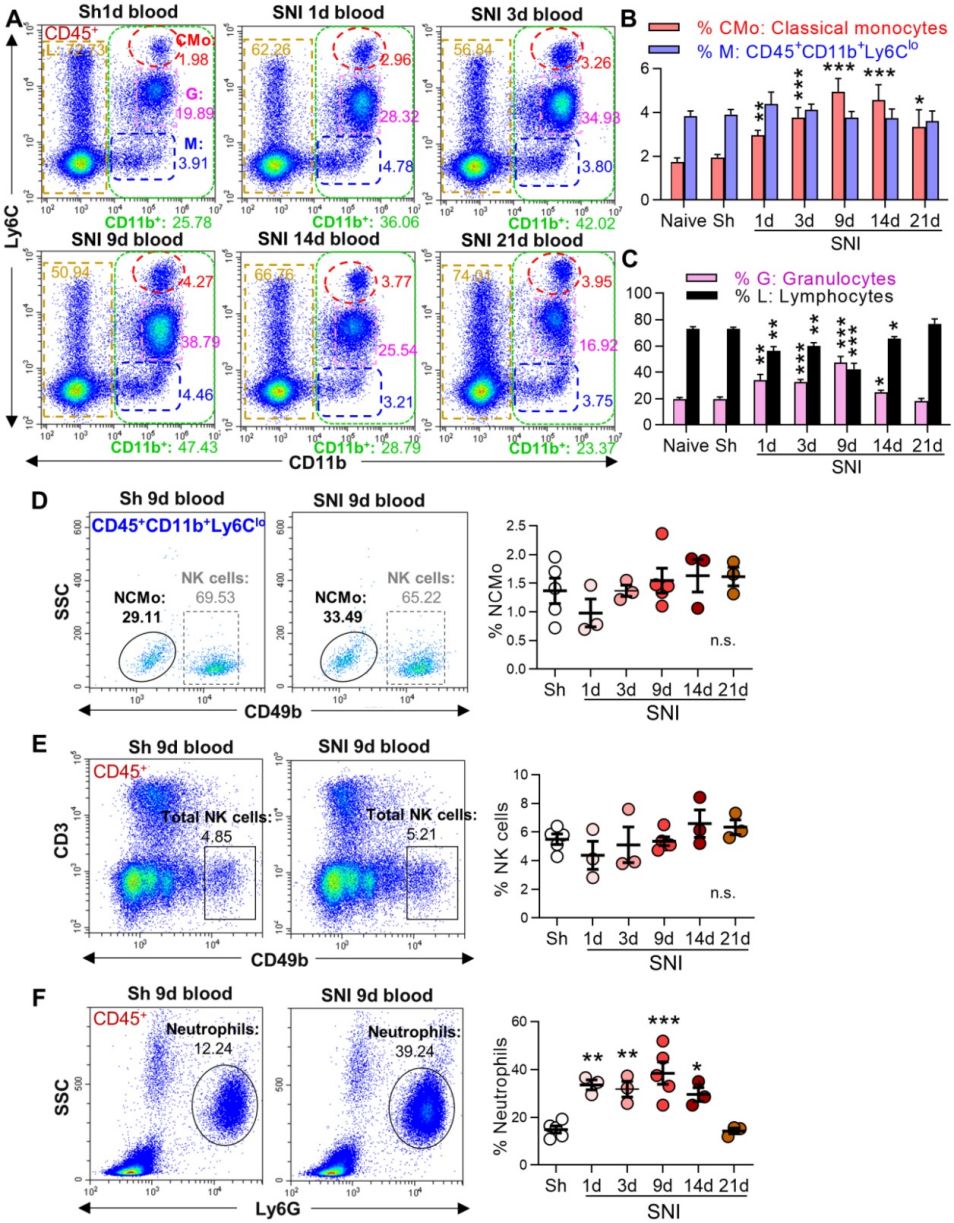
** SNI causes a persistent increase in circulating classical monocytes.** (**A**) Representative flow cytometry profiles of different subtypes of leukocytes gated by CD45^+^ in sham (Sh) and SNI mice. The inserted number shows the percentages of the classical monocytes (CMo: CD45^+^CD11b^+^L6C^high^, red), CD45^+^CD11b^+^L6C^low^ cells (M, blue), granulocytes (G: CD45^+^CD11b^+^L6C^med^, purple) and lymphocytes (L: CD45^+^CD11b^-^, golden) in all CD45^+^ leukocytes. Sh: sham group, S1d, S3d, S9d, S14d, S21d show the groups harvested at different days after SNI. (**B, C**) Percentages of blood classical monocytes (CMo), CD45^+^CD11b^+^L6C^low^ cells (M), granulocytes (G) and lymphocytes (L) in all leukocytes (CD45^+^) from different groups. n = 10 for naïve and S9d groups. In sham mice, the experiments were performed at three time points (1 d, 3 d, 9 d after sham operation, 3 mice at each time point). As no difference was detected, the data from sham mice were used together; n = 4 in other groups. (**D-F**) Changes in nonclassical monocytes (NCMo: CD45^+^CD11b^+^Ly6C^low^CD49b^-^, C), NK cells (CD45^+^CD3^-^CD49b^+^, D), and neutrophils (CD45^+^SSC^med^Ly6G^+^, F) in sham and SNI groups (n = 3-5 mice/group). *P < 0.05, **P < 0.01, ***P < 0.001, and n.s.: not significant, *vs.* sham group, two-way ANOVA with Bonferroni's post hoc test used for B and C, one-way ANOVA with Bonferroni's post hoc test used for D-F.

To investigate whether the cross-talk between peripheral immune cells and the brain may be involved in neuroinflammation and cognitive deficit in neuropathic pain, we first analyzed circulating leukocytes in sham and SNI male mice by flow cytometry. After erythrocyte removal, we gated different leukocyte subtypes with corresponding cell markers (Figure S1), and then examined their changes induced by SNI. Compared to naive and sham groups, the percentage of myeloid subsets (CD11b^+^ CD45^+^ 21, 30, Figure 1A, green boxes) increased from day 1 after SNI, and the change persisted for at least 14 days. Further analysis revealed that the proportion of classical monocytes (CMo: CD45^+^CD11b^+^Ly6C^high^, red circles) increased on day 1, peaked on day 9, and was still higher on day 21 after SNI than in naïve or sham mice (Figure 1A, B). Granulocytes (G: CD45^+^CD11b^+^Ly6C^med^, purple boxes) were also substantially increased from day 1 to day 9 after SNI, while lymphocytes (L: CD45^+^CD11b^-^, golden boxes) were significantly decreased (Figure 1A, C). Both granulocytes and lymphocytes returned to baselines on day 14 or 21 after SNI. The cells with CD45^+^CD11b^+^Ly6C^low^ (M, Figure 1A, blue boxes) were NK cells and nonclassical monocytes (Figure S1 C, D), which did not change at different time points after SNI (Figure 1D, E). The proportion of neutrophils increased from day 1 to day 14 after SNI compared to sham mice (Figure 1F). Of note, similar changes in circulating leukocyte subpopulations were also observed in female SNI mice (Figure S3A, B).

### SNI increases brain perivascular macrophages without affecting BBB

**Figure 2:**
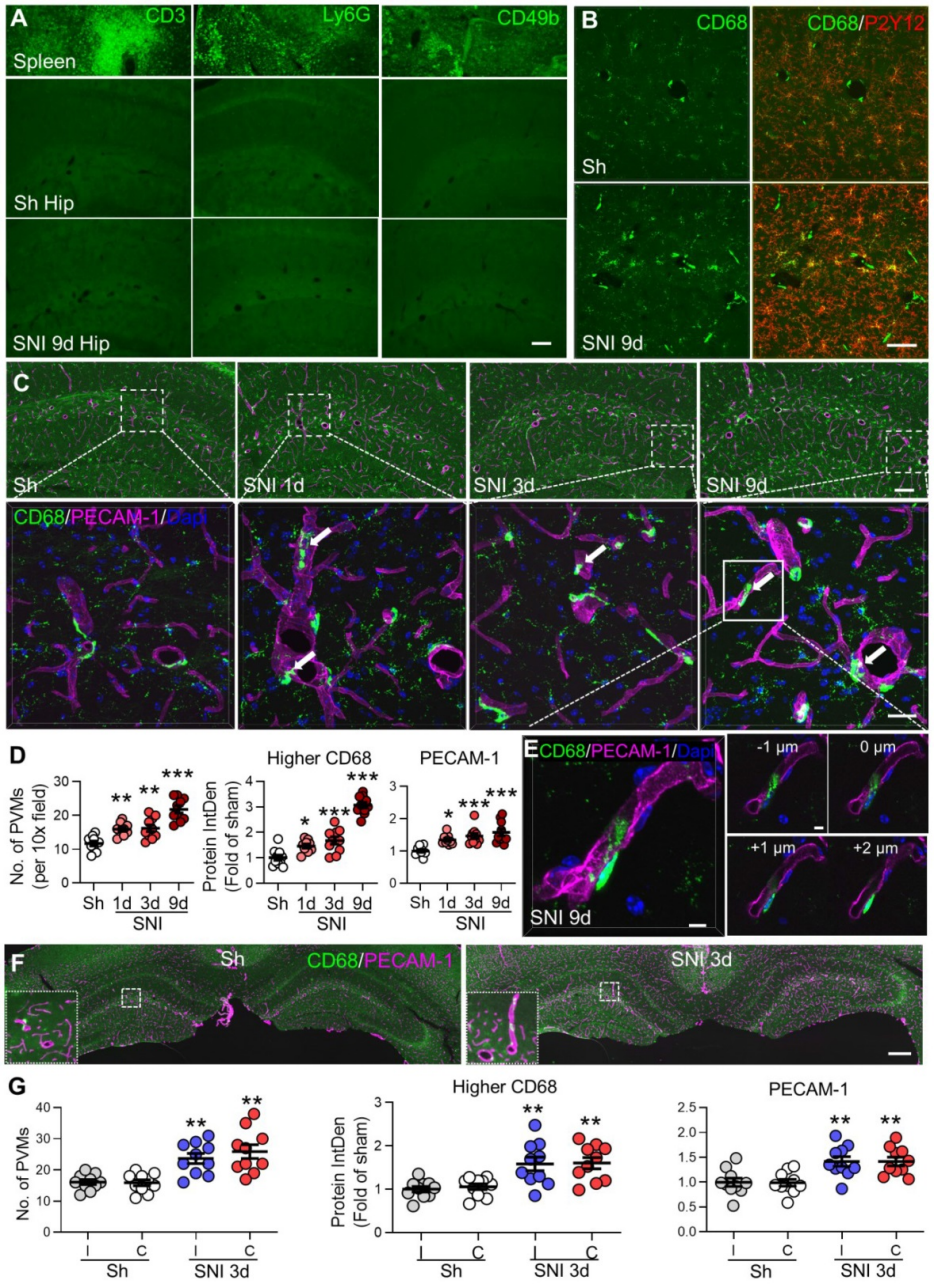
** SNI increases perivascular macrophages (PVMs) in the bilateral hippocampi.** (**A**) Representative micrographs of CD3 (T lymphocytes marker), Ly6G (neutrophils marker) or CD49b (NK cells marker) staining of hippocampal sections from sham (Sh) or SNI 9 d groups and spleen sections (positive controls). Scale bar: 100 µm. (**B**) Expression of CD68 and P2Y12 in the hippocampal sulcus on day 9 after sham or SNI surgery. P2Y12^+^ cells with weaker CD68 staining are microglia (yellow) and with stronger CD68 staining (CD68^high^) are PVMs located predominantly in perivascular spaces (green). Scale bar: 50 µm. (**C**, **D**) Numbers of PVMs (CD68^high^, green) and the relative integrated density (IntDen) of CD68^high^ or PECAM-1 (magenta) in the hippocampus with time after SNI compared to the sham group. Arrows indicate monocytes in the process of migrating into the perivascular space. Scale bars, 100 µm and 25 µm (bottom). n = 3 mice/group, 3-4 slices/mice. (**E**) Triple-staining confocal merge image (left) and multiple plane images (right) of the CD68^high^ monocyte and endothelium (marked by PECAM-1) at 1 µm intervals showing monocyte migration across the endothelium into the perivascular space. Scale bar = 5 µm. (**F, G**) Changes of CD68^high^ cells and PECAM-1 in the bilateral hippocampi in sham and SNI 3 d groups (n = 3 mice/group, 3-4 images/mice). I: ipsilateral hippocampus, C: contralateral hippocampus. Scale bar: 250 µm. * P < 0.05, **P < 0.01, ***P < 0.001 *vs.* sham group, one-way ANOVA with Bonferroni's post hoc test.

We first examined immune cell changes in the hippocampus in sham and SNI male mice by immunostaining to investigate if the increased peripheral leukocytes may infiltrate into the brain. We found that CD3^+^ lymphocytes, Ly6G^+^ neutrophils and CD49b^+^ NK cells could hardly be detected in the hippocampus (Figure 2A), while CD68^+^ cells were observed in hippocampal perivascular spaces in sham and SNI mice (Figure 2B). It has been reported that CD68 is expressed in monocytes, macrophages, and microglia 31 while P2Y12 is exclusively expressed in microglia in the CNS 32. We performed double staining with CD68 and P2Y12 to distinguish macrophages and microglia. Monocytes within the blood vessels had been removed by PBS super-fusion during sample preparation. As reported previously 33, the ramified CD68^low^ cells, which are P2Y12^+^ and widely distributed in the hippocampus, are microglia. We observed that the rod-shaped CD68^high^ cells, which were P2Y12^-^ and located near the endothelium marked by platelet endothelial cell adhesion molecule (PECAM-1, also known as CD31) in the perivascular spaces, were perivascular macrophages (PVMs, Figure 2C-E). Compared to the sham group, the PVM number and intensity of CD68^high^ signal around the hippocampal sulcus were substantially increased starting from day 1 and reaching approximately 2-3 times higher on day 9 after SNI. In parallel, PECAM-1 expression was also upregulated from day 1 and peaked on day 9 after SNI. Moreover, we observed a few monocytes that exhibited morphological features typical for adhering or migrating to the vasculature (Figure 2C, arrows). The multiple focal planes revealed a rod-shaped CD68^high^ cell with a mono-lobed nucleus in the perivascular space while still maintaining a long cytoplasmic process inside the vasculature, which appeared to be migrating through the vascular endothelium into the perivascular space (Figure 2E). The increase in PVMs and PECAM-1upregulation were observed in the bilateral hippocampi after SNI (Figure 2F, G). SNI also enhanced the number of PVMs in female mice (Figure S3C).

We analyzed CD68^high^ cells located in perivascular spaces in whole brain slices to determine if SNI may also increase PVMs in other brain regions. Increased numbers of PVMs were detected in primary somatosensory cortex (S1), retrosplenial granular cortex. (RSGc) and thalamus (Thal) with the most obvious change in the hippocampus (Figure S4A-C).

To test if the increase in PVMs induced by SNI might interrupt BBB, we i.v. injected Evans blue or NaFlu and examined the hippocampus as there are abundant blood vessels around hippocampal fissure vulnerable to pathological challenges 34, 35. We found that BBB permeability was not disrupted by SNI (Figure S5A, B). Furthermore, the protein and mRNA levels of two key tight junction proteins, occludin and zona occludens 1 (ZO-1), in the hippocampus at varying times after SNI were not different from those in sham-operated mice (Figure S5C, D).

### Transmigration of circulating monocytes contributes to the increase in perivascular macrophages following SNI

**Figure 3:**
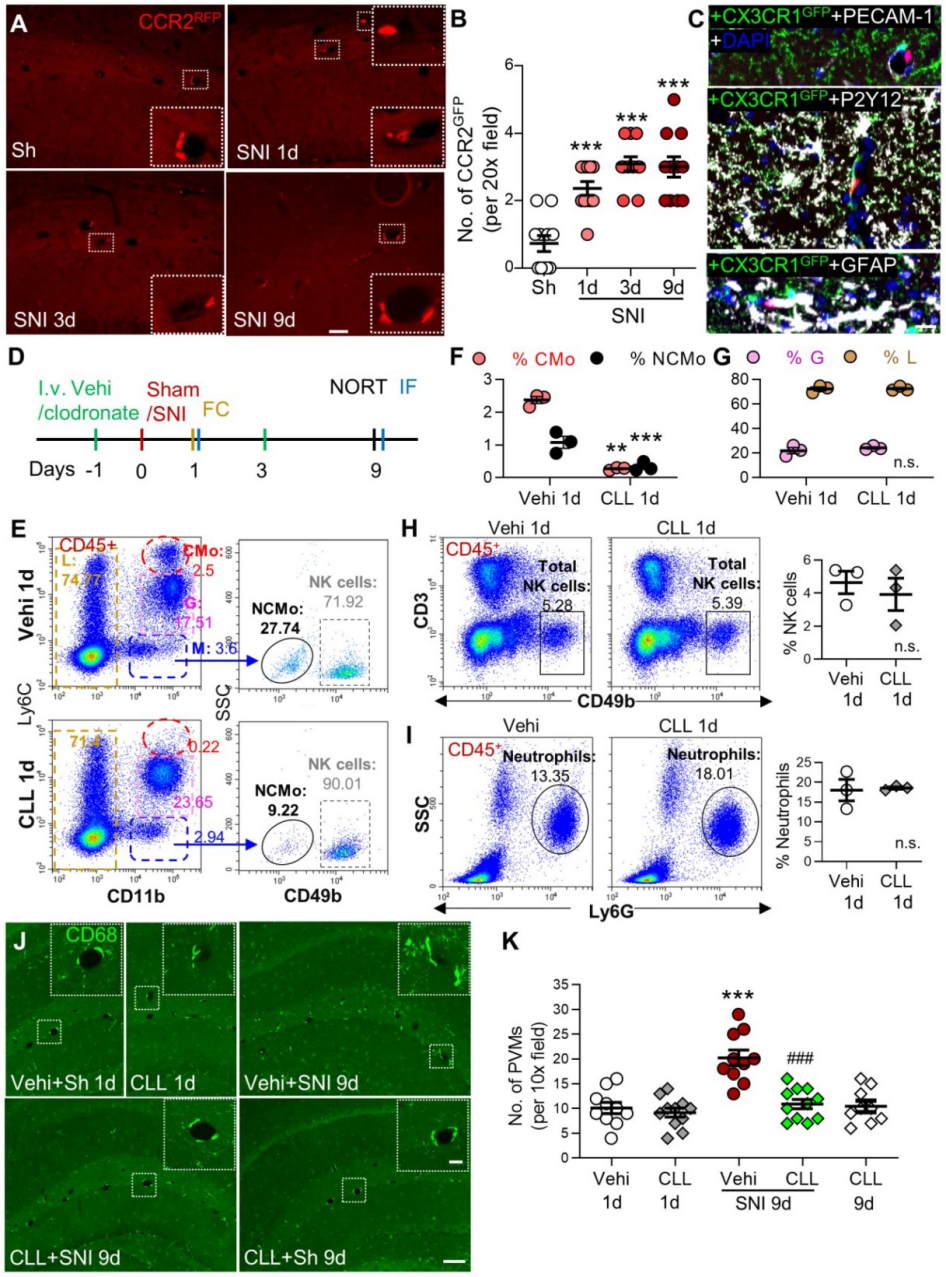
** Circulating inflammatory monocytes contribute to increased PVMs in the hippocampus in neuropathy.** (**A**, **B**) CCR2^RFP/+^ monocytes (red) in sham (Sh) and SNI 1d, 3d, 9d groups (n = 3-4 slices/mice, 3 mice/group). The insets in A are magnified from the white dotted boxes. (**C**) Triple-staining confocal merged image showing the relationship of a CCR2^RFP^ cell (red) with the endothelium (marked by PECAM-1, white), microglia (marked by CX3CR1^GFP^, green and P2Y12, white), astrocytes (GFAP, white) and nucleus (DAPI, blue). Scale bars: 200 µm (A) and 20 µm (C). **(D)** Diagram showing the experimental procedure. Vehicle (Vehi) or clodronate liposomes (CLL, 15 mL/kg, i.v.) were injected on 1 d before and 3 d after sham or SNI surgery. The efficacy of CLL on monocytes was verified at 18 h after first injection of CLL by blood flow cytometry (FC) and brain tissue immunofluorescence (IF). The short-term memory (STM) index analyzed with the novel object recognition test (NORT) and IF was performed 9 d after surgery. (**E-I**) Percentages of different circulating leukocyte subpopulations at 1 d after injection of vehicle or clodronate (n = 3 for each group). (**J, K**) PVM (CD68^high^) numbers around the hippocampal sulcus from different groups (n = 3-4 slices/mice, 3 mice/group). The insets were magnified from the white dotted boxes. Scale bars: 100 µm in F, 20 µm in insets. **P < 0.01, ***P < 0.001 *vs.* vehicle or sham group, ^###^*P* < 0.001 *vs.* vehicle SNI group, one-way ANOVA with Bonferroni's post hoc test (B, K), two-way ANOVA with Bonferroni's post hoc test (F, G), and two-tailed Student's *t-*test (H, I).

Based on our data that classical monocytes in blood and PVMs in the brain were persistently increased, we tested if PVMs might be derived from circulating monocytes. We used double-transgenic CCR2^RFP/+^ and CX3CR1^GFP/+^ mice, in which resident CX3CR1-positive microglia were labeled with GFP and the circulating CCR2-positive monocytes with RFP and RFP/GFP 36. The CCR2^RFP/+^ cells were observed in the perivascular spaces of the hippocampal fissure in both sham and SNI mice (Figure 3A). The number of the perivascular CCR2^RFP/+^ cells increased with time after SNI compared to the sham group (Figure 3A, B). High-resolution triple-staining confocal images showed that CCR2^RFP/+^ cells were located near the endothelial cells labeled with PECAM-1, and were not co-localized with the microglia marker P2Y12/CX3CR1^GFP/+^ or astrocyte marker GFAP (Figure 3C). The data suggested that circulating monocytes transmigrated to the brain and became PVMs in neuropathic pain. To confirm this, we depleted circulating monocytes by intravenous injection of clodronate liposomes (CLL, 15 mL /kg) on day 1 before and day 3 after SNI (Figure 3D). Consistent with previous studies 24, 37, the clodronate injections depleted the majority of classical (CMo, CD11b^+^Ly6C^high^) and nonclassical (NCMo, CD11b^+^Ly6C^-^) monocytes on day 1 after the injection in naïve mice, but did not affect lymphocytes including NK cells or neutrophils (Figure 3E-I). Importantly, we found that CLL injection reliably prevented the PVMs (CD68^high^) increase induced by SNI, but did not affect the number of PVMs in naïve mice (Figure 3J, K).

### Depletion of circulating monocytes abolishes gliosis memory and deficit induced by SNI

**Figure 4:**
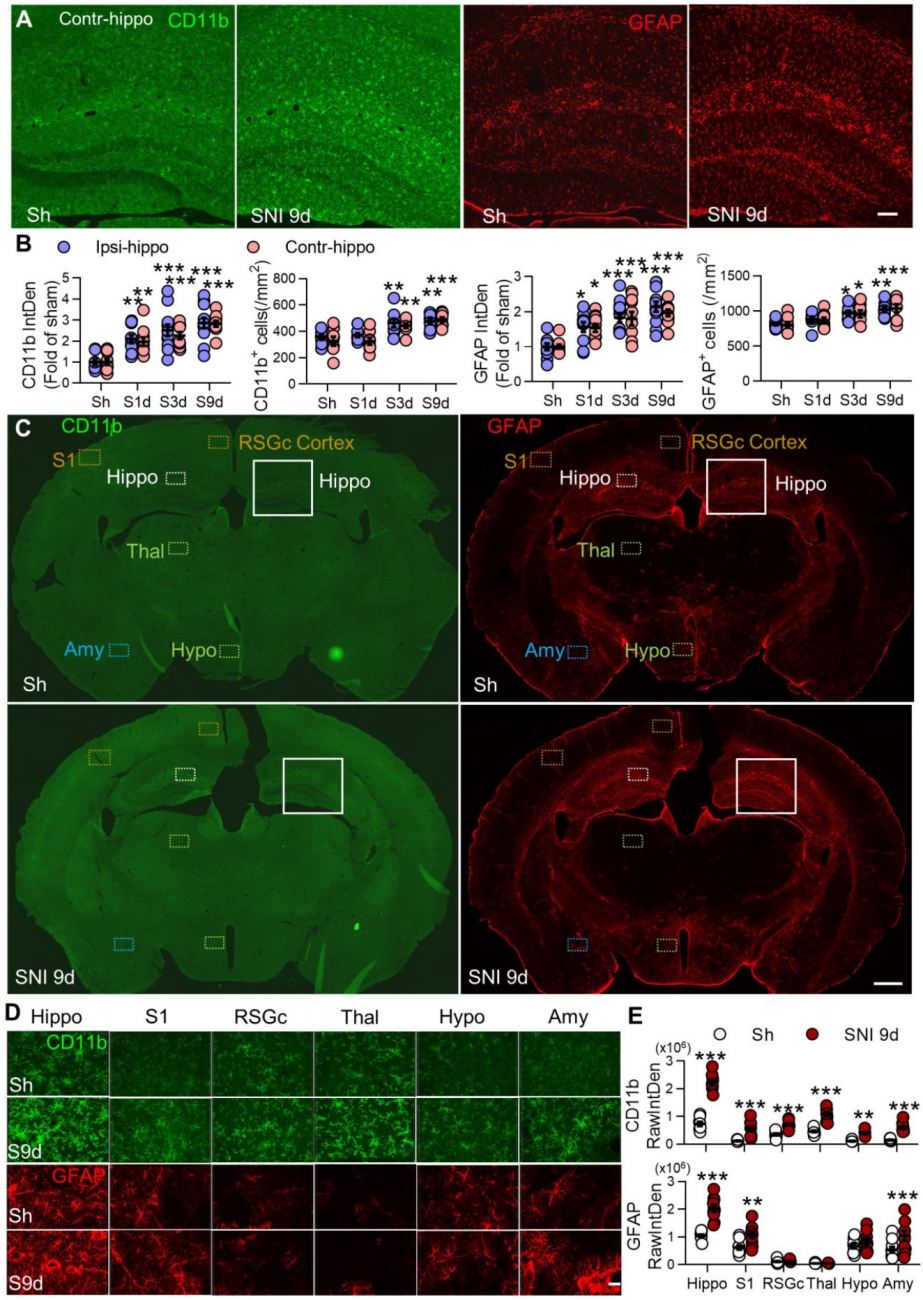
** SNI causes progressive gliosis in many brain regions, especially in bilateral hippocampi**. (**A**, **B**) Temporal change of the morphology, normalized integrated density (IntDen) of CD11b and GFAP, and numbers in microglia and astrocytes in bilateral hippocampi at 1, 3, and 9 d after SNI. (**C-E**) Fluorescent IntDen of CD11b and GFAP in different brain regions as indicated in sham and SNI groups at 9 d after surgery. CD11b- and GFAP- immunostaining micrographs in both groups are from the same section. The white line boxed areas in C are magnified in A to show the morphological changes of glial cells. The images in D are magnified from dotted boxes in C. RawIntDen analysis of CD11b and GFAP staining was used for statistics in E. Hip: hippocampus, S1: primary somatosensory cortex, RSGc: retrosplenial granular cortex, Thal: Thalamus, Hypo: hypothalamus, Amy: amygdala. Scale bars: 100 µm (A), 500 µm (C) and 25 µm (D). n = 3 mice/group, 3-4 images/mice, *P < 0.05, **P < 0.001, ***P < 0.001 *vs.* sham group, two-way ANOVA with Bonferroni's post hoc test.

We found that both microglia (marked by CD11b) and astrocytes (marked by GFAP) were progressively activated in bilateral hippocampi from day 1 to day 9 after unilateral SNI, especially around the hippocampal sulcus (Figure 4A, B). SNI also induced gliosis in the hippocampus of female mice (Figure S3D, E). Furthermore, SNI activated glial cells in many other brain regions on day 9 after SNI, and the change was most profound in the hippocampus (Figure 4C-E). The spatial and temporal pattern of gliosis was similar to the change of PVMs after SNI (Figure S4).

**Figure 5:**
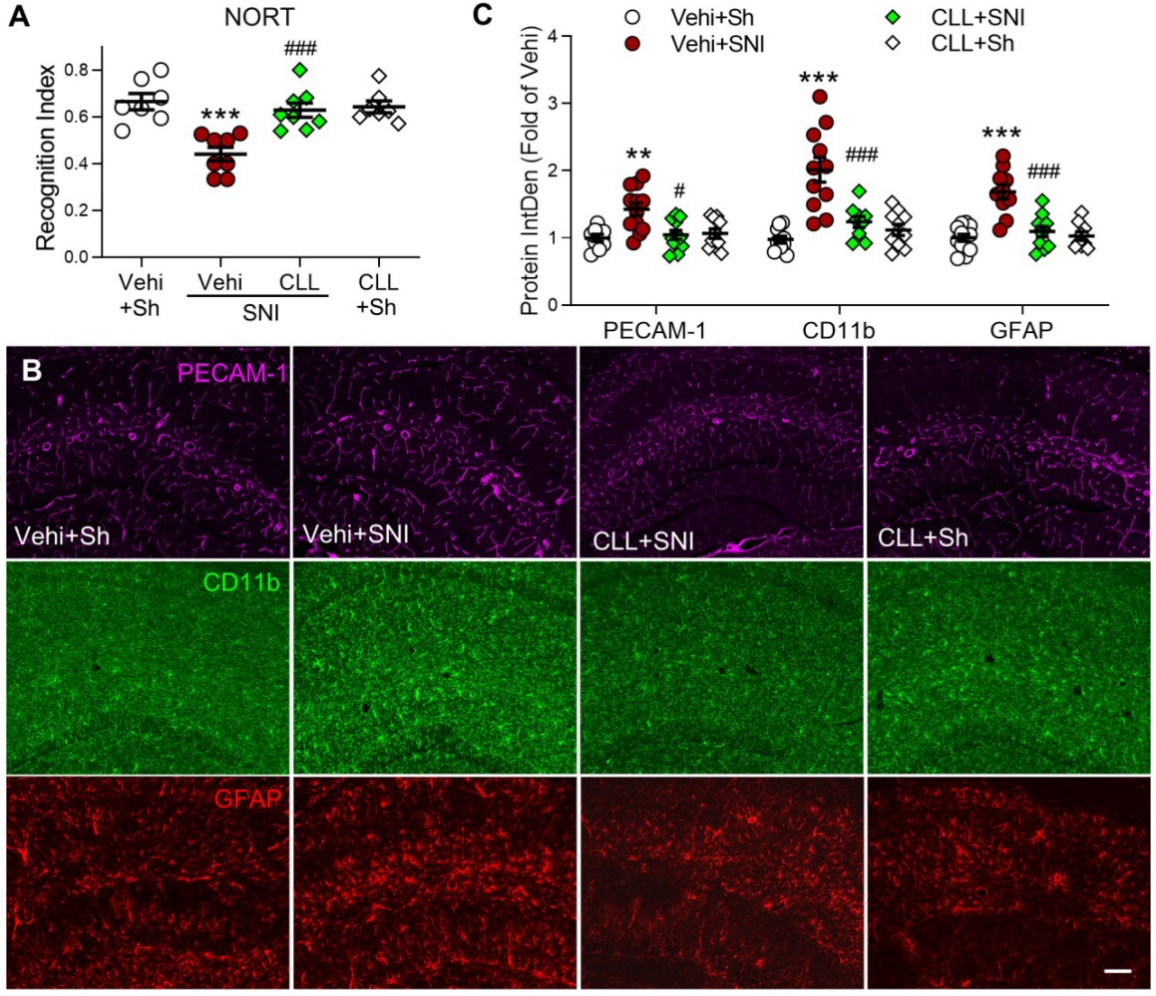
** Depletion of circulating monocytes abolishes memory deficit and gliosis induced by SNI** (**A**) Recognition index accessed by NORT in mice in the sham group (Sh) and in groups 9 d after SNI with clodronate or vehicle (Vehi) injection are shown (*n* = 7 in vehicle and CLL sham groups, n = 8 in other groups). (**B, C**) Effect of clodronate on PECAM-1 expression and activation of microglia (CD11b) and astrocytes (GFAP) at 9 d after SNI. Scale bar = 100 µm. n = 3 mice/group, 3-4 images/mice, **P < 0.01, ***P < 0.001 *vs.* vehicle sham group, ^##^*P* < 0.01, ^###^*P* < 0.001 *vs.* vehicle SNI group, one-way ANOVA with Bonferroni's post hoc test (A) and two-way ANOVA with Bonferroni's post hoc test (C).

To investigate the possibility that gliosis may result from PVM recruitment in SNI mice, we injected CLL, which completely blocked SNI-induced increase in PVMs (Figure 3J, K), and was capable of preventing the memory decline and hippocampal gliosis induced by SNI. Indeed, the manipulation significantly prevented the STM decline (Figure 5A) and SNI-induced upregulation of PECAM-1, CD11b and GFAP in the hippocampus (Figure 5B, C). These data suggested that circulating monocyte recruitment into perivascular space may induce cognitive decline by activating glial cells.

### SNI enhances CXCL12 in plasma and perivascular spaces

**Figure 6:**
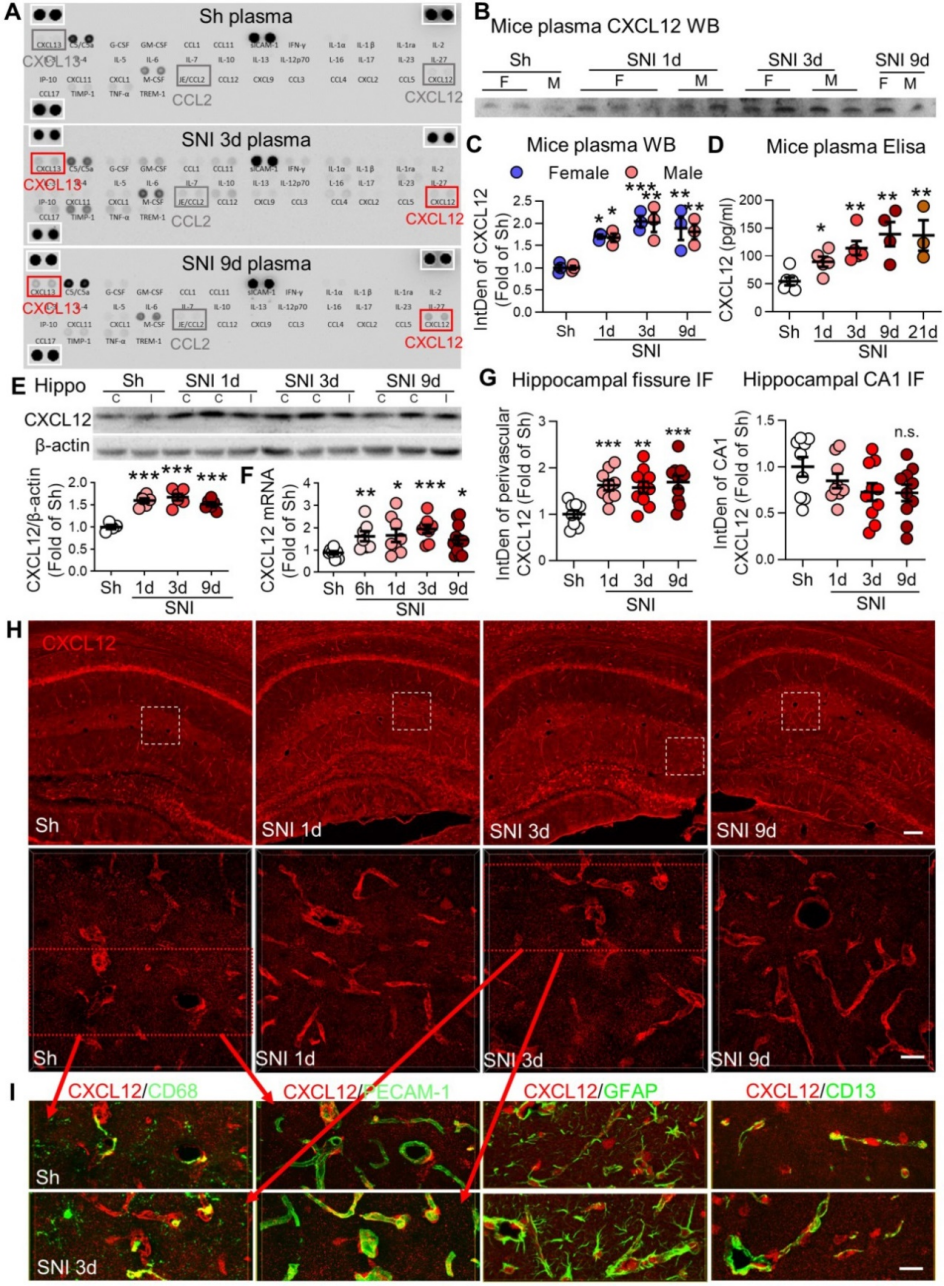
** SNI produces persistent elevation of CXCL12 in plasma and hippocampal perivascular spaces.** (**A**) Cytokine array results showing changes in plasma cytokines and chemokines in sham (Sh) and in 3 d, 9 d after SNI groups. There was no difference in the positive control protein levels (white boxes) among the three groups. (**B, C**) Plasma CXCL12 expression in different groups of female (F) and male (M) mice. n = 3 mice/group. (**D**) ELISA results showing changes in plasma CXCL12 concentrations with time after SNI, compared to sham mice (n = 3-6 mice/group). (**E**) SNI induced upregulation of CXCL12 in bilateral hippocampi (n = 4-6 mice/group). I: ipsilateral hippocampus, C: contralateral hippocampus. (**F**) CXCL12 mRNA in bilateral hippocampi at different time points after SNI (n = 7-12 mice/group). (**G, H**) Expression of CXCL12 around the hippocampal sulcus but not in the CA1 area was upregulated from 1 d to 9 d after SNI compared to sham mice (n = 3-4 slices, 3 mice/group). The white dotted boxes in top images are magnified below. Scale bars: 100 µm (top) and 25 µm (below). n.s.: no significant difference, *P < 0.05, **P < 0.01, ***P < 0.001 *vs.* sham group, two-way ANOVA with Bonferroni's post-hoc test used for **C** and one-way ANOVA with Bonferroni's post-hoc test used for D-G. (**I**) Multiple sets of images show the colocalization of CXCL12 (red) with the PVM marker CD68 (green), endothelial cell marker PECAM-1 (green), astrocyte marker GFAP (green) or pericyte maker CD13 (green) in sham and SNI 3d groups. Red arrows showing the images in I are magnified from the red boxes in H. Scale bar = 25 µm.

To explore the possible mechanisms underlying monocyte recruitment into the brain perivascular space, we first used an array (blot) of 40 different cytokines and chemokines to analyze their changes in plasma induced by SNI. Compared to sham mice, CXCL12 and CXCL13 were persistently upregulated for at least 9 days after SNI, whereas other cytokines and chemokines were only transiently elevated in SNI mice (Figure 6A). We focused on CXCL12 as this chemokine is believed to mediate monocyte unidirectional migration by activation of CXCR4 38, while CXCL13 does not have a known role in monocyte trafficking 39. Western blotting confirmed that plasma CXCL12 was significantly increased on day 1, peaked on day 3, and maintained the high level on day 9 after SNI in both male and female mice (Figure 6B, C). ELISA results further showed that plasma CXCL12 level was gradually increased from day 1 to 21 after SNI (Figure 6D). Interestingly, we found that both CXCL12 protein and mRNA were significantly upregulated in bilateral hippocampi from 1 to 9 d following SNI (Figure 6E, F). Immunofluorescence staining showed that CXCL12 in hippocampal sulcus but not in the CA1 region was upregulated (Figure 6G, H), indicating the change was associated with the abundance of blood vessels. Double staining revealed that CXCL12 was colocalized with CD68^high^, endothelial cell marker PECAM-1, astrocyte marker GFAP, and pericyte marker CD13 on day 3 after SNI (Figure 6I). Also, we found that CLL injection, which deleted the majority of circulating monocytes, blocked CXCL12 upregulation in the hippocampus (Figure S6). Thus, circulating monocytes were needed for CXCL12 upregulation induced by SNI.

### Blockade of CXCL12-CXCR4 signaling prevents SNI-induced behavioral and cellular changes

**Figure 7:**
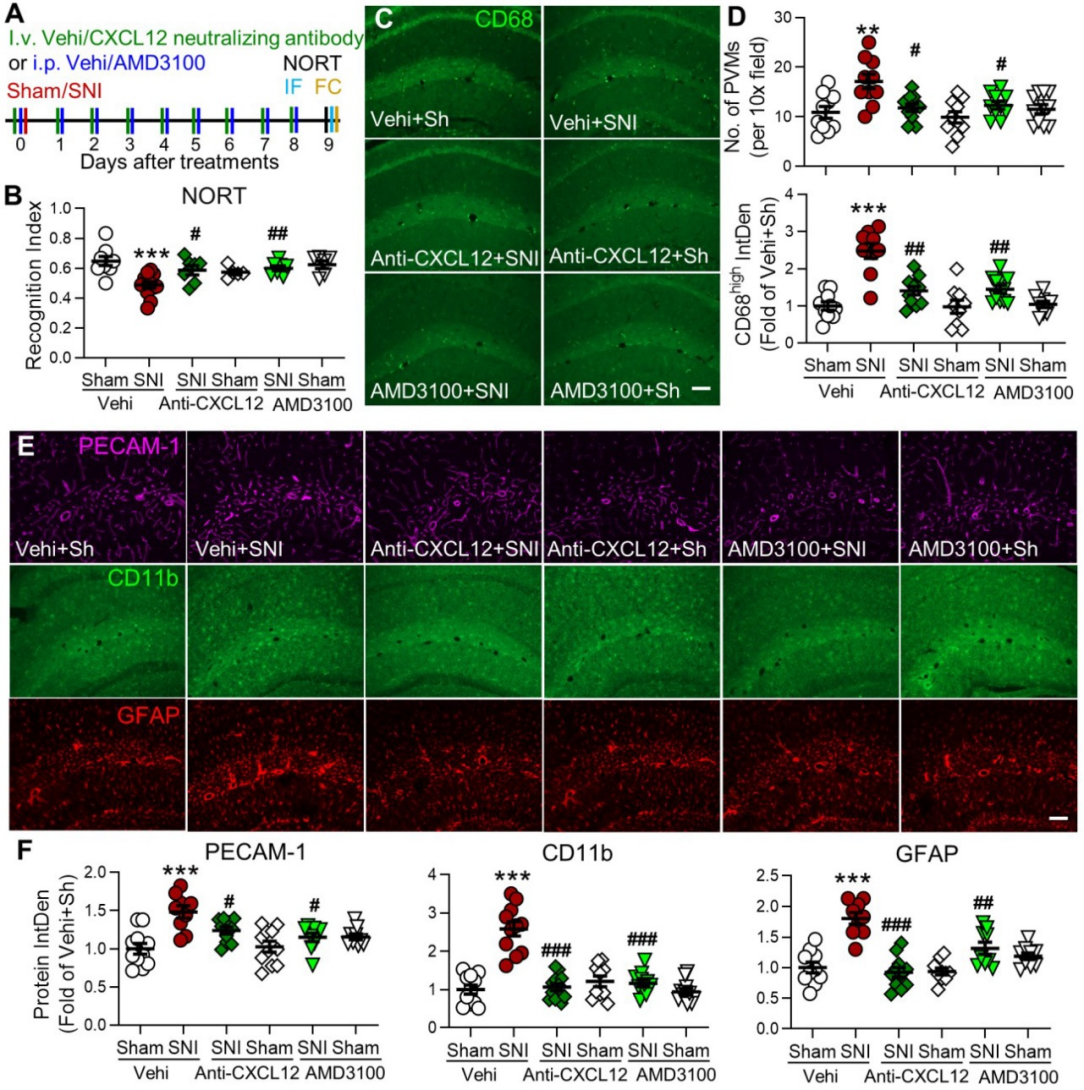
** Blocking the CXCL12-CXCR4 pathway reverses SNI-induced cognitive impairment, PVMs increase, and gliosis in the hippocampus.** (**A**) Experimental protocol showing that anti-CXCL12 neutralizing antibody (20 ng/200 µL, i.v.) or CXCR4 antagonist AMD3100 (200 µg/mL, 1 mg/kg, i.p.) or vehicle (Vehi) was applied 30 min before and daily after sham (Sh) or SNI for 9 successive days. On day 9 after the injection, memory function was analyzed with NORT, and mice were perfused for IF and FC. (**B**) Anti-CXCL12 neutralizing antibody or AMD3100 injection prevented SNI-induced decline in the recognition index but had no effect in sham mice (n =5-12 mice/group). (**C-F**) Number of PVMs (CD68^high^) and the IntDen of CD68^high^, PECAM-1, CD11b, and GFAP in the hippocampus in indicated groups. Scale bar = 100 µm. n = 3 mice/group, 3-4 slices/mice. *P < 0.05, **P < 0.01, ***P < 0.001 *vs.* vehicle sham group, ^#^*P* < 0.05, ^##^*P* < 0.01, ^###^*P* < 0.001 *vs.* vehicle SNI group, one-way ANOVA with Bonferroni's posthoc test.

So far, we showed that SNI-induced memory decline was associated with the increase in PVMs and gliosis in many brain regions and the persistent upregulation of CXCL12 in both plasma and hippocampus. To investigate the causal link between CXCL12 upregulation and changes induced by SNI, CXCL12 neutralizing antibody (20 ng/200 μL, i.v.) or CXCR4 antagonist AMD3100 (200 μg/mL, 1 mg/kg, i.p.) 26, 40, 41 was injected 30 min before and daily for 9 successive days after SNI (Figure 7A). We found that both treatments prevented the cognitive index decline in in SNI mice, but had no effect in sham mice (Figure 7B). The PVM increase and PECAM-1, CD11b, and GFAP upregulation in hippocampal sulcus induced by SNI were also prevented by either anti-CXCL12 antibody or AMD3100 administration (Figure 7C-F). AMD3100 also prevented the increase in classical monocytes, granulocytes, and neutrophils and the decrease in blood lymphocytes in SNI mice but not in sham mice (Figure S7). These results indicated that the CXCL12-CXCR4 pathway was necessary to transform circulating monocytes into PVMs, induction of neuroinflammation, and subsequent development of memory deficit in SNI mice.

### Elevation of plasma CXCL12 is sufficient to induce gliosis and memory deficit

**Figure 8:**
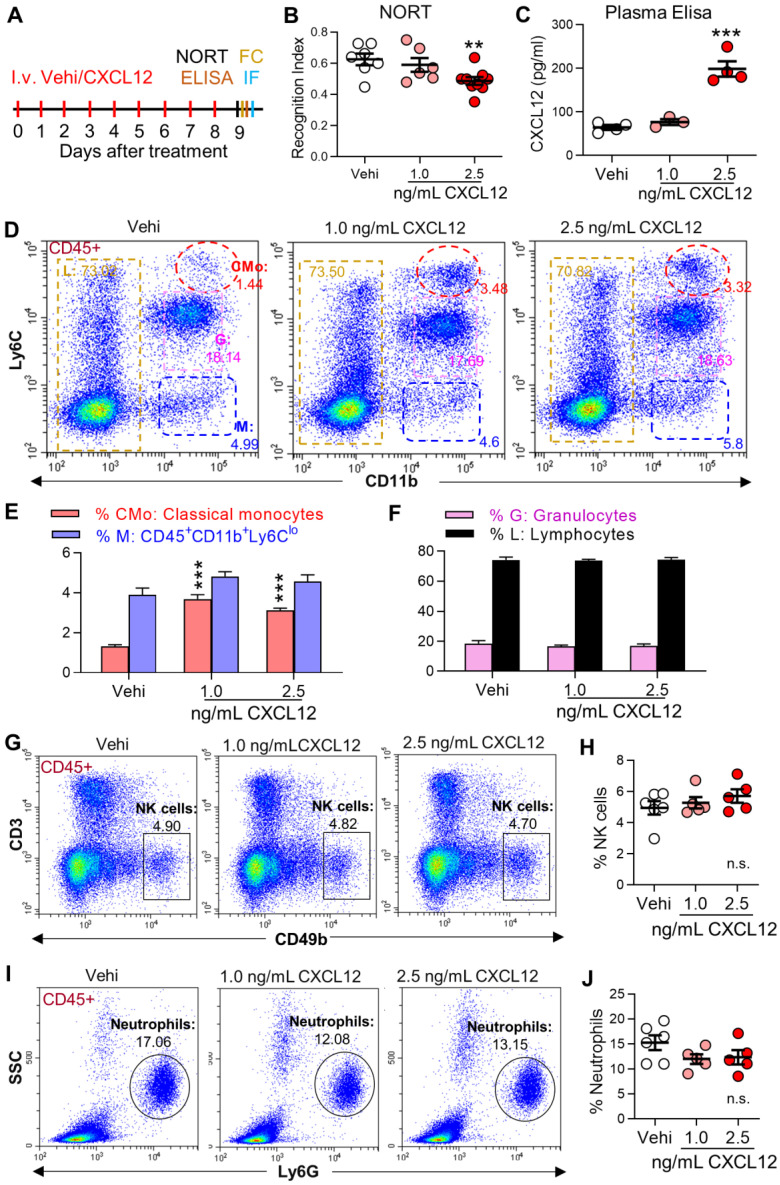
** Intravenous injection of CXCL12 induces memory deficit and elevates circulating monocytes and plasma CXCL12.** (**A**) CXCL12 (1.0 and 2.5 ng/mL, 200 µL) or same volume of vehicle (Vehi) was injected for successive 9 days via the tail vein of naïve mice, and memory function was analyzed by NORT. The blood and brain tissue were harvested for ELISA, FC, and IF after the behavioral test. (**B**) Effects of injection of vehicle or different dosages of CXCL12 on STM index are shown (6-9 mice/group). (**C**) ELISA results revealed that CXCL12 injection at 2.5 ng/mL but not 1.0 ng/mL for 9 days elevated plasma CXCL12 (3-4 mice/group). The plasma CXCL12 was measured 24 h after the last injection of CXCL12. (**D-J**) Effects of iv injection of CXCL12 at 1.0 and 2.5 ng/mL on circulating leukocyte subpopulations. n = 5-6 mice/group. **P < 0.01, ***P < 0.001 vs. vehicle group, one-way ANOVA with Bonferroni's post hoc test (B, C, H, J) and two-way ANOVA with Bonferroni's post hoc test (E, F).

**Figure 9:**
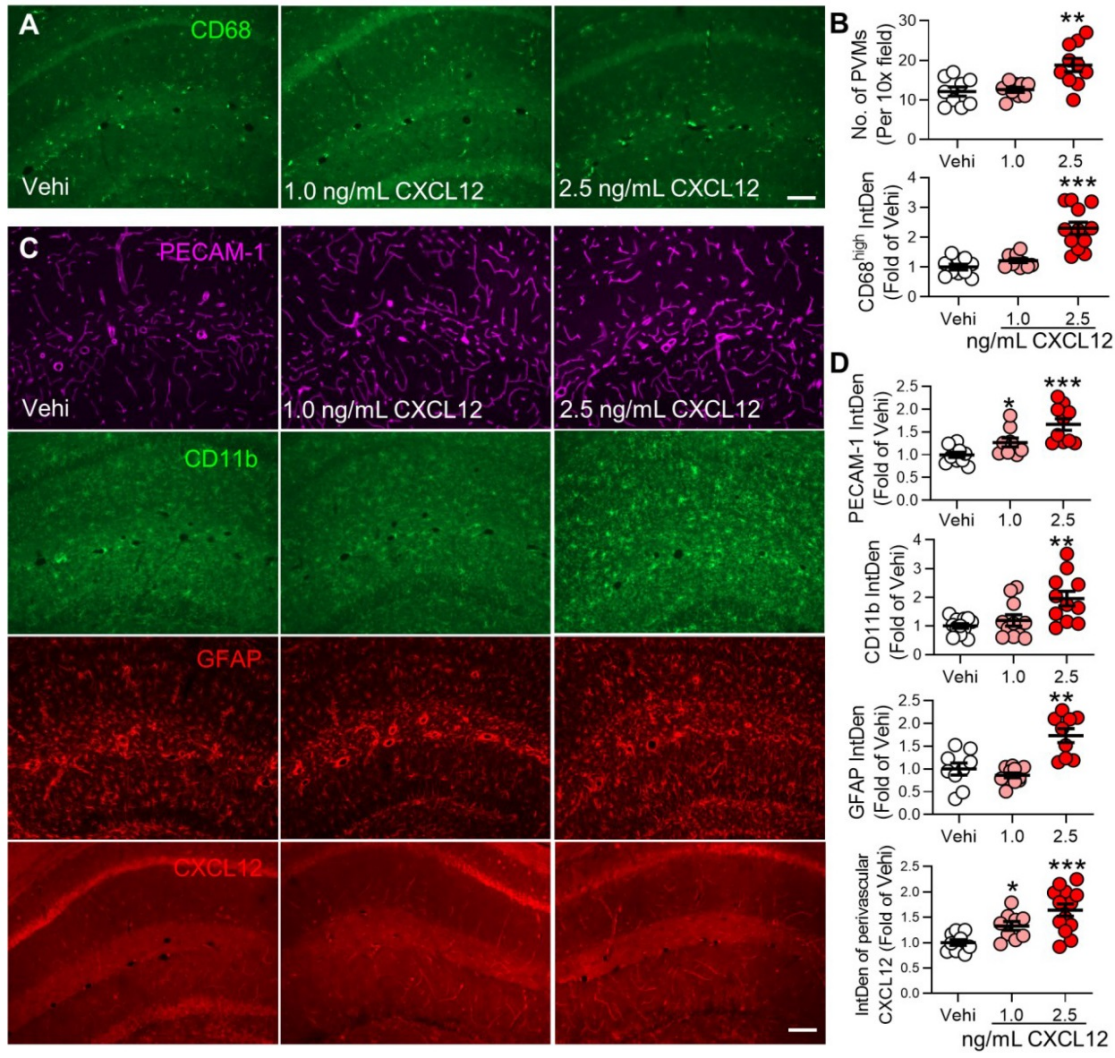
** CXCL12 intravenous injection increases perivascular macrophages and PECAM-1 expression and gliosis in the hippocampus.** (**A**, **B**) Number of PVMs (CD68^high^) and immune fluorescence intensities of CD68^high^ in the hippocampal perivascular space in vehicle and CXCL12 groups (n = 3 mice, 3-4 slices /mice). (**C, D**) Effect of vehicle or CXCL12 injection on the CD11b, GFAP, PECAM-1, and CXCL12 expression. n = 3-4 slices/mice, 3 mice/group. Scale bar = 100 µm. ^*^P < 0.05, ^**^P < 0.01, ^***^P < 0.001 *vs.* vehicle group, one-way ANOVA with Bonferroni's post-hoc test.

To determine if the persistent increase in plasma CXCL12 observed in SNI mice is sufficient to induce behavioral, cellular, and molecular changes induced by SNI, CXCL12 was i.v. injected into naïve mice for 9 successive days (Figure 8A). Compared to the vehicle group, CXCL12 at 2.5 ng/mL but not at 1.0 ng/mL reduced recognition index (Figure 8B) and elevated plasma CXCL12 (Figure 8C). However, CXCL12, at both 1.0 ng/mL and 2.5 ng/mL, enhanced classical monocytes but affected neither CD45^+^CD11b^+^L6C^low^ cells, lymphocytes, granulocytes (neutrophils) nor NK cells in the blood (Figure 8D-J). In the hippocampus, CXCL12 at 2.5 ng/mL, but not at 1.0 ng/mL, increased PVMs and immune fluorescence density of CD68^high^ (Figure 9A, B). The upregulation of GFAP and CD11b was only detected in the high dose (2.5 ng/mL) group, while PECAM-1 and CXCL12 upregulations were evident with both doses (Figure 9C, D). The data suggested that a threshold plasma CXCL12 level is required to trigger a positive feedback loop that maintains a continuous CXCL12 overproduction sufficient to recruit monocytes and activate glial cells, leading to memory deficit.

### Chronic pain patients with memory deficit display notable increases in monocytes and plasma CXCL12

**Figure 10:**
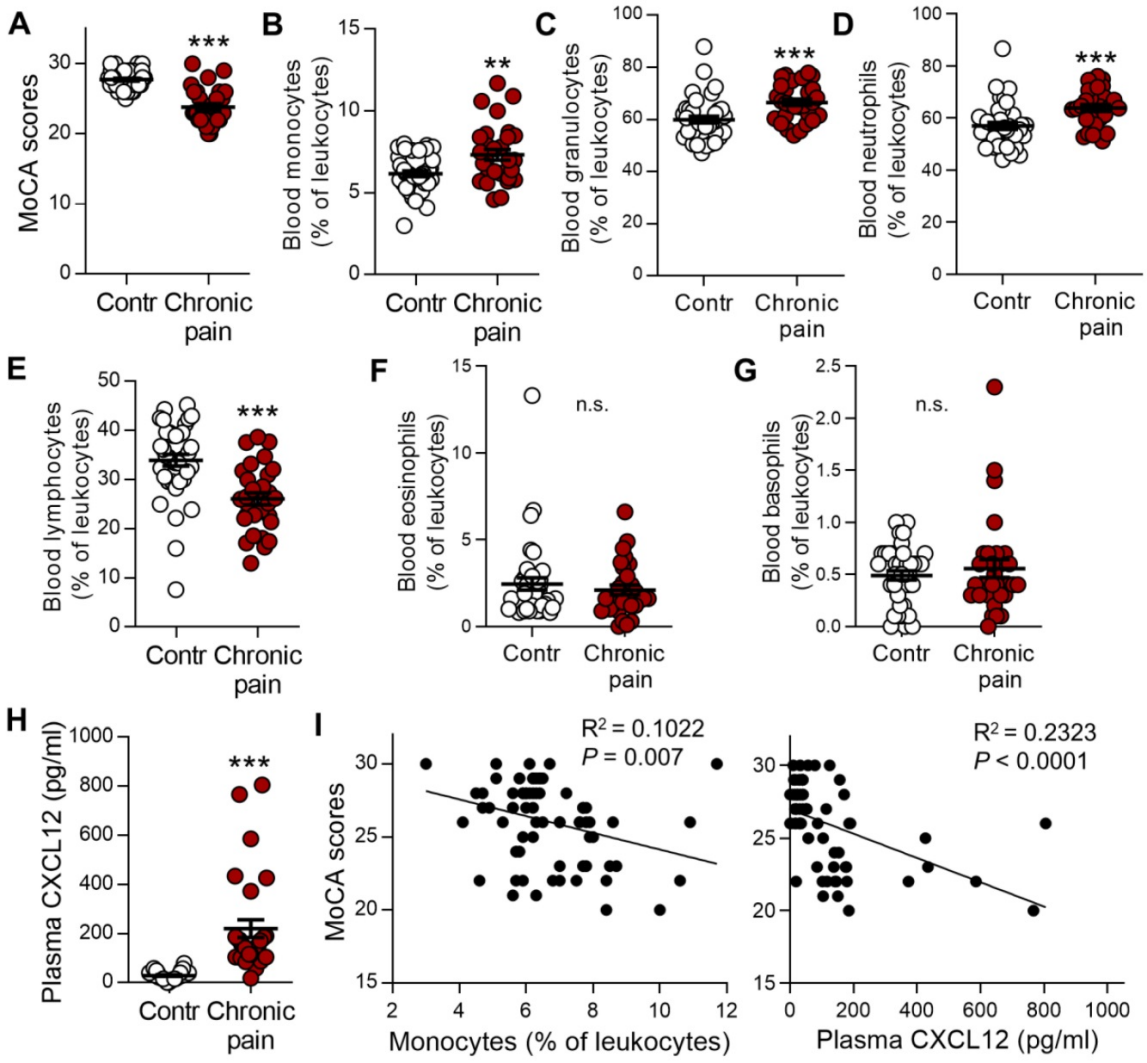
** Circulating monocytes and plasma CXCL12 are increased in patients with chronic pain and are correlated with cognitive decline.** (**A**) Memory function accessed by MoCA in patients with chronic neuropathic pain (Chronic pain, n = 30) was lower than healthy controls (Contr, n = 40). (B-G) Percentages of monocytes (**B**), granulocytes (**C**), neutrophils (**D**), lymphocytes (**E**), eosinophils (**F**), and basophils (**G**) in various groups were determined by routine blood analysis. (**H**) Concentration of plasma CXCL12 in healthy control subjects and chronic pain patients are shown. ^**^P < 0.01, ^***^P < 0.001 *vs.* healthy control group, two-tailed Student's *t-*test. (**I**) Scatterplots showing that the percentage of circulating monocytes (Spearman rank correlation, R^2^ = 0.1022, *P* = 0.007) and plasma CXCL12 concentration (R^2^ = 0.2323,* P* < 0.0001) was negatively correlated with MoCA scores in healthy controls and patients with chronic pain.

To determine whether murine data are comparable with the human condition, we analyzed the impact of blood immune cells and CXCL12 levels on the memory function measured by Montreal Cognitive Assessment (MoCA). Patients with chronic low back pain, neuralgia, or radiculopathy were compared with gender-, age-, and education-matched healthy controls (Table 1). Consistent with previous human studies 4, 42, 43, the mean value of MoCA scores was significantly lower in chronic pain patients than in healthy controls (23.8 ± 0.5 vs 27.7 ± 0.2, *P* < 0.001, n = 30, 40/group, Figure 10A). The percentages of monocytes and granulocytes (neutrophils) in the total leukocytes were significantly higher (Figure 10B-D) while that of lymphocytes was lower in chronic pain patients than in healthy controls (Figure 10E). There was no difference in eosinophils and basophils between chronic pain patients and normal controls (Figure 10F, G). Furthermore, plasma CXCL12 level was significantly higher in chronic pain patients compared to healthy controls (Figure 10H, 220.11 ± 35.72 pg/mL vs 29.44 ± 2.68 pg/mL, *P* < 0.001). Correlation analysis using Spearman rank correlation revealed that the percentage of circulating monocytes or plasma CXCL12 concentration was negatively correlated with MoCA scores (Figure 10I). These results were consistent with SNI mice data.

## Discussion

The present study revealed that circulating classical monocytes were persistently increased following SNI. Some of these monocytes transmigrated into brain perivascular spaces and were converted to PVMs, which are implicate in brain neuroinflammation and associated memory deficit. CXCL12 upregulation in plasma and in perivascular spaces is critical for transformation of monocytes into PVMs. Importantly, we found that chronic pain patients also exhibited increased circulating monocytes and plasma CXCL12 correlated with cognitive impairment. Our study provides new insight into mechanisms underlying cognitive deficit associated with chronic pain. Targeting the circulating CXCL12 and monocytes may alleviate the cognitive deficit, a key component of high-impact chronic pain 6.

### Critical role of PVMs in neuroinflammation and memory deficit in neuropathic pain

Previous studies have shown that the infiltration of immune cells into brain parenchyma resulting from the BBB interruption leads to cognitive deficit in a variety of pathological conditions 44-46. However, we found monocyte transmigration into perivascular spaces without immune cell infiltration into brain parenchyma and BBB interruption. This is consistent with a previous study that partial ligation of the sciatic nerve does not affect BBB 25.

The origin of PVMs is still debatable with some studies showing that PVMs are derived from peripheral monocytes and others reporting that they are not replenished by circulating monocytes in normal conditions (see 47 for a review). Several findings in our study suggest that at least some PVMs are derived from circulating monocytes in neuropathic pain: 1) Circulating classical monocytes (Figure 1A, B) and the PVMs, (CD68highP2Y12- rod-shaped and located near the endothelium in perivascular spaces), were persistently increased in SNI mice (Figure 2B-D). 2) Using double-transgenic CCR2^RFP/+^ and CX3CR1^GFP/+^ mice and double-staining technique, we showed that at least some of the PVMs were derived from monocytes (Figure 3A-C). And 3) Deletion of circulating monocytes prevented the increase in PVMs induced by SNI mice (Figures 2C-E and 3J, K). Recent studies indicated that monocytes do not infiltrate into the spinal dorsal horn after peripheral nerve injury 48, 49. Thus, circulating monocytes may not cross the blood-brain/spinal barrier following peripheral nerve injury, but stay in perivascular spaces to induce neuroinflammation and promote chronic pain and memory deficit.

Previous studies reported that glial activation in the brain is more robust in the contralateral side after peripheral nerve injury 50, 51. However, our data showed that the unilateral SNI resulted in gliosis in many brain regions, and the change was most profound in bilateral hippocampi. This observation was consistent with our previous study that unilateral SNI blocked long-term potentiation in bilateral hippocampi 1. Whereas, the question remains as to how unilateral SNI leads to the widespread gliosis in brain. We found that the increase in PVMs was in a spatial and temporal fashion similar to gliosis in SNI mice. Obstruction of PVM recruitment by either deleting circulating monocytes or blocking CXCL12-CXCR4 signaling prevented gliosis and memory decline induced by SNI, indicating that the increase in monocyte transmigration into PVMs results in widespread gliosis induced by SNI. The brain neuroinflammation induced by peripheral nerve injury may be initiated at and distributed along the blood vessels. Our observation that the increase in PVMs and gliosis were most obvious in the hippocampus may simply be due to abundant blood vessels around the hippocampal fissure vulnerable to pathological challenges 34, 35.

A recent study reported that cognitive deficit measured with the Morris water maze and novel object location test were seen only in male mice, but not in female mice in the L5 spinal nerve transection model of neuropathic pain 52. While our current study showed that the SNI-induced pathological changes, including increase in circulating leukocytes (Figures 1A, B and S3A, B), brain PVMs (Figures 2, S3C, and S4), and gliosis (Figures 4 and S4D, E), the elevation of plasma CXCL12 (Figure 6A-D) and SNI-induced cognitive impairment were not different between male and female mice. The difference in neuropathic pain models may contribute to this dissimilarity.

### CXCL12 overproduction is critical for the recruitment of perivascular macrophages in neuropathy

SNI induces changes in many chemokines that recruit monocytes. We hypothesized that CXCL12 plays a key role in monocyte transformation to PVMs in the brain in neuropathic pain, based on the following evidence: 1) CXCL12 was consistently elevated in mouse plasma from day 1 after SNI, lasting for at least 21 days (Figure 6A-D), and it was also raised in plasma of human patients with chronic pain (Figure 10H). 2) SNI-induced CXCL12 upregulation was detected in almost all cell types of perivascular spaces in the hippocampus (Figure 6I), in which PVM increase and gliosis were most profound. 3) Significantly, the SNI-induced CMo and PVM increase, hippocampal gliosis, and memory decline were prevented by blockade of CXCL12-CXCR4 signaling (Figures 7 and S7), and mimicked by iv injection of CXCL12 (Figures 8 and 9). Thus, CXCL12 upregulation is necessary and sufficient for monocyte transformation into PVMs.

We investigated the mechanism underlying the continual upregulation of CXCL12 in blood and perivascular spaces. A previous study has shown that blood monocytes secrete CXCL12 and express CXCR4 and CXCR7 53, contributing to the persistent increase of CXCL12 in plasma via a positive autocrine feedback mechanism. Herein, we showed that iv injection of CXCL12 led to its increased level in hippocampal perivascular spaces (Figure 8C), while the deletion of monocytes prevented the CXCL12 upregulation in hippocampus induced by SNI (Figure S6). These data indicated that a positive feedback may also exist between blood and the brain.

Furthermore, we showed that in naïve mice the increase in circulating classical monocytes was induced by low (1.0 ng/mL) and high doses (2.5 ng/mL) of CXCL12 injection. On the other hand, the increase in plasma CXCL12 and PVMs, gliosis, and memory decline were only induced by high dose CXCL12 (Figures 8 and 9), indicating the key role of upregulated CXCL12 in neuroinflammation. The data suggested that a threshold level of plasma CXCL12 is required to trigger the positive feedback loop and maintain continuous CXCL12 overproduction, sufficient to recruit monocytes and activate glial cells, leading to memory deficit.

Previous studies have shown that inflammatory cytokines, including IL-1β and TNF-α, are substantially elevated in plasma or cerebral spinal fluid 1, 18. Cytokines can cross the BBB by saturable transport systems 54-56 and upregulate CXCL12 57, 58. Thus, SNI-induced upregulation of inflammatory cytokines may also play a role in the initiation and maintenance of CXCL12 overproduction. Furthermore, we found that the SNI-induced upregulation of PECAM-1 in the hippocampus was prevented by blocking CXCL12 (Figure 7E, F) and mimicked by CXCL12 injection (Figure 9C, D). As CXCL12 plays an important role in monocyte transmigration under inflammatory conditions 59, 60, it may also recruit monocytes into perivascular spaces by upregulation of PECAM-1. Another CXCL12 receptor, CXCR7, has been implicated in CXCL12-regulated monocyte function, survival, differentiation into macrophages 61, 62, and brain function 63, and may also be involved in the monocyte transformation into PVMs.

In summary, the circulating classical monocytes and plasma CXCL12 were enhanced in neuropathic mice and in human chronic pain patients. The memory decline resulting from the neuroinflammation was driven by the recruitment of inflammatory monocytes into perivascular space. Peripheral monocytes and plasma CXCL12 may serve as biomarkers and therapeutic targets for cognitive impairments associated with high-impact chronic pain.

## Supplementary Material

Supplementary figures and tables.

## Figures and Tables

**Table 1:** Characteristics of human participants

	Control (n=40)	Chronic pain (n=30)	*P* value (group effect)
Gender, males/females	16/24	14/16	-
Age (years)	48.8±1.6	53.9±3.1	0.1148
Education (years)	10.0±0.4	9.9±0.6	0.9089
Disease duration (months)	N/A	43.7±17.7	-
Pain index-NRS	N/A	6.0±0.2	-

N/A: not applicable. Data are expressed as the number of patients, mean, and SEM. The *t-*test was used to compare variables between two groups.

## Abbreviations

CXCL12: C-X-C motif chemokine 12
SNI: Spared nerve injury
PVMs: Perivascular macrophages
PECAM-1: platelet endothelial cell adhesion molecule-1
BBB: blood-brain barrier
NORT: Novel object recognition test
IF: Immunofluorescence
MoCA: Montreal Cognitive Assessment
