# CXCL12-mediated monocyte transmigration into brain perivascular space leads to neuroinflammation and memory deficit in neuropathic pain: Erratum

**DOI:** 10.7150/thno.92433

**Published:** 2023-11-28

**Authors:** Chun-Lin Mai, Zhi Tan, Ya-Nan Xu, Jing-Jun Zhang, Zhen-Hua Huang, Dong Wang, Hui Zhang, Wen-Shan Gui, Jun Zhang, Zhen-Jia Lin, Ying-Tong Meng, Xiao Wei, Ying-Tao Jie, Peter M. Grace, Long-Jun Wu, Li-Jun Zhou, Xian-Guo Liu

**Affiliations:** 1 Department of Physiology and Pain Research Center, Zhongshan School of Medicine, Sun Yat-sen University, Guangzhou 510080, China.; 2 Guangdong Province Key Laboratory of Brain Function and Disease, Guangzhou 510080, China.; 3 Department of Anesthesiology and Pain Clinic, the First Affiliated Hospital of Sun Yat-Sen University, Guangzhou, 510080, China.; 4 Division of Emergency Medicine, the First Affiliated Hospital of Sun Yat-Sen University, Guangzhou, 510080, China.; 5 Department of Clinical Laboratory, The First Affiliated Hospital, Sun Yat-sen University, Guangzhou, 510080, China.; 6 Department of Anesthesiology, Guangdong Second Provincial General Hospital, Guangzhou, 510317, China.; 7 Department of Critical Care & Respiratory Care Research (PMG), University of Texas MD Anderson Cancer Center, Houston, Texas, USA.; 8 Department of Neurology, Mayo Clinic, Rochester, MN 55905, USA.

In the original Figure 7E of our article, the authors mistakenly reused the GFAP immunostaining representative image of the Vehi+Sham group for the anti-CXCL12+Sham group. This is because an error occurred in converting the original purple fluorescence of GFAP for each group to red fluorescence during preparation of representative pictures of Figure 7E. Unfortunately, this error was not found in multiple revisions. The correct version of Figure 7E is shown below. The correction made in this Erratum does not affect the results and conclusions in this study. The authors apologize for any inconvenience the errors may have caused.

## Figures and Tables

**Figure 7:**
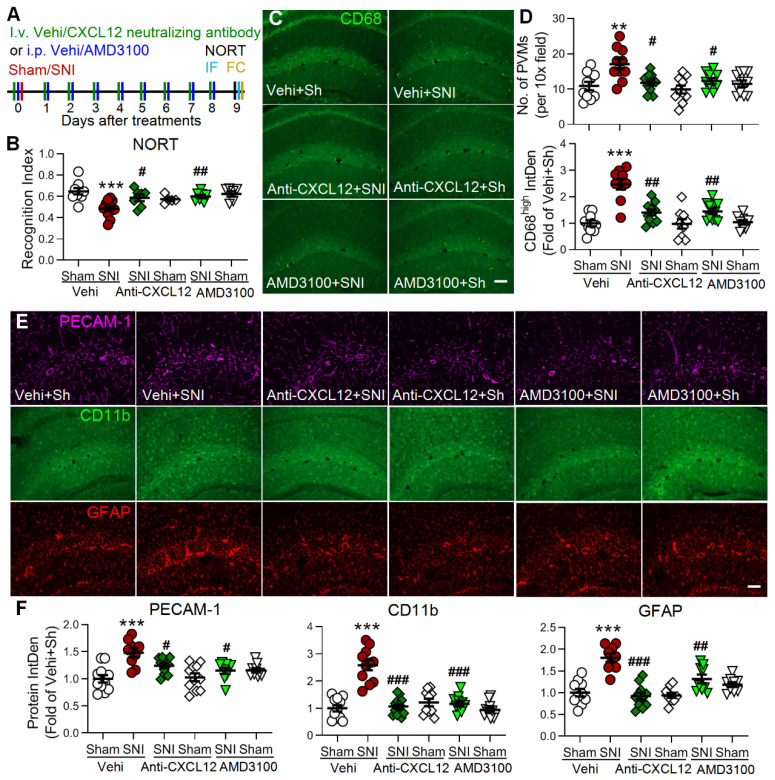
Blocking the CXCL12-CXCR4 pathway reverses SNI-induced cognitive impairment, PVMs increase, and gliosis in the hippocampus. (A) Experimental protocol showing that anti-CXCL12 neutralizing antibody (20 ng/200 μl, i.v.) or CXCR4 antagonist AMD3100 (200 μg/ml, 1 mg/kg, i.p.) or vehicle (Vehi) was applied 30 min before and daily after sham (Sh) or SNI for 9 successive days. On day 9 after the injection, memory function was analyzed with NORT, and mice were perfused for IF and FC. (B) Anti-CXCL12 neutralizing antibody or AMD3100 injection prevented SNI-induced decline in the recognition index but had no effect in sham mice (n =5-12 mice/group). (C-F) Number of PVMs (CD68^high^) and the IntDen of CD68^high^, PECAM-1, CD11b, and GFAP in the hippocampus in indicated groups. Scale bar = 100 μm. n = 3 mice/group, 3-4 slices/mice. *P < 0.05, **P < 0.01, ***P < 0.001 *vs.* vehicle sham group, ^#^*P* < 0.05, ^##^*P* < 0.01, ^###^*P* < 0.001 *vs.* vehicle SNI group, one-way ANOVA with Bonferroni's posthoc test.

