# Supplementary material for: CXCL12-mediated monocyte transmigration into brain perivascular space leads to neuroinflammation and memory deficit in neuropathic pain
Source: Theranostics. 2021 Jan 1;11(3):1059–78. doi: 10.7150/thno.44364 (PMC7738876; doi:10.7150/thno.44364)
Supplement: Supplementary file 1 — Supplementary figures and tables. [file thnov11p1059s1.pdf]

# CXCL12-mediated monocyte transmigration into brain perivascular space leads to neuroinflammation and memory deficits in neuropathic pain

Chun-Lin Mai[1$], Zhi Tan[1$], Ya-Nan Xu[1$], Jing-Jun Zhang[3$], Zhen-Hua Huang[4], Dong Wang[5], Hui Zhang[6], Wen-Shan Gui[1], Jun Zhang[1], Zhen-Jia Lin[1], Ying-Tong Meng[1], Xiao Wei[1], Ying-Tao Jie[1], Peter M. Grace[7], Long-Jun Wu[8], Li-Jun Zhou[1, 2]✉, Xian-Guo Liu[1, 2]

[1]Department of Physiology and Pain Research Center, Zhongshan School of Medicine, Sun Yat-sen University, Guangzhou 510080, China

[2]Guangdong Province Key Laboratory of Brain Function and Disease, Guangzhou 510080, China

[3]Department of Anesthesiology and Pain Clinic, the First Affiliated Hospital of Sun Yat-Sen University, Guangzhou, 510080, China

[4]Division of Emergency Medicine, the First Affiliated Hospital of Sun Yat-Sen University, Guangzhou, 510080, China

[5]Department of Clinical Laboratory, The First Affiliated Hospital, Sun Yat-sen University, Guangzhou, 510080, China

[6]Department of Anesthesiology, Guangdong Second Provincial General Hospital, Guangzhou, 510317, China

[7]Department of Critical Care & Respiratory Care Research (PMG), University of Texas MD Anderson Cancer Center, Houston, Texas, USA.

[8]Department of Neurology, Mayo Clinic, Rochester, MN 55905, USA

[$] These authors contributed equally to this work.

✉Corresponding author: Li-Jun Zhou, E-mail: zhoulij3@mail.sysu.edu.cn.

# Supporting Figures

## Figure S1

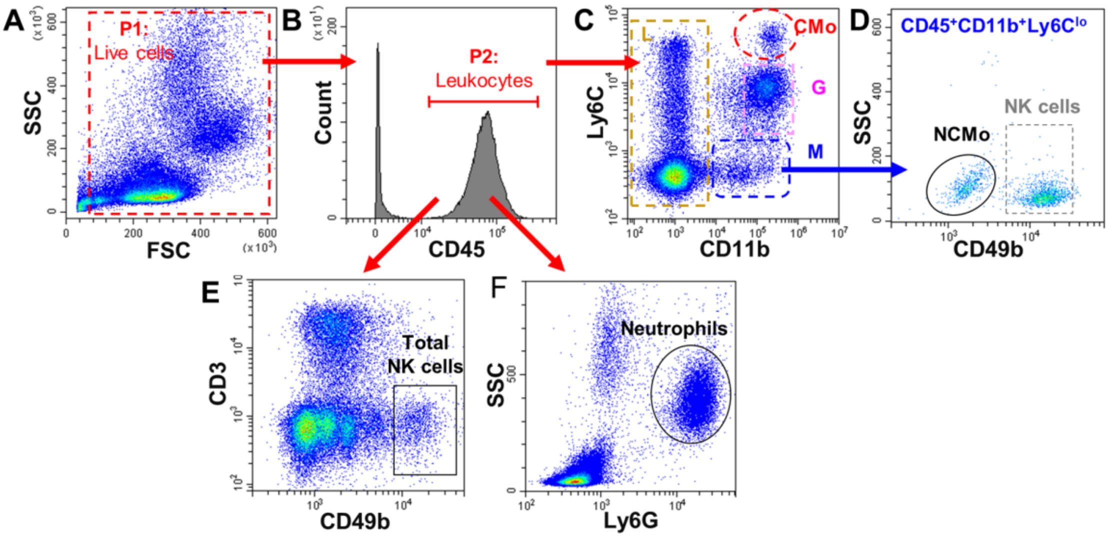


**Figure S1. Gating strategy for circulating leukocyte subtypes.** (**A, B**) After erythrocyte removal, blood live cells were first gated on forward and side scatter (FSC, SSC, A, P1), and then gated with CD45 antibody (B, P2) for isolating leukocytes. (**C, D**) The leukocytes were first subdivided into classical monocytes (CMo: $CD45^{+}CD11b^{+}Ly6C^{high}$, red box), granulocytes (G: $CD45^{+}CD11b^{+}Ly6C^{med}$, purple box) and lymphocytes (L: $CD45^{+}CD11b^{-}$, golden box) based on CD11b and Ly6C staining. $CD45^{+}CD11b^{+}Ly6C^{low}$ (M, blue box) cells were further divided into NK cells ($CD45^{+}CD11b^{+}Ly6C^{low}CD49b^{+}$, grey box) and nonclassical monocytes (NCMo: $CD45^{+}CD11b^{+}Ly6C^{low}CD49b^{-}$, black circle) based on CD49b staining. (**E, F**) Representative plots pre-gated on $CD45^{+}$ cells (B, P2) from which further subsets were divided into total NK cells (E, $CD45^{+}CD3^{-}$ $CD49b^{+}$) based on CD3 and CD49b expression or neutrophils (F, $CD45^{+}SSC^{med}Ly6G^{+}$) based on SSC and Ly6G as indicated.

## Figure S2

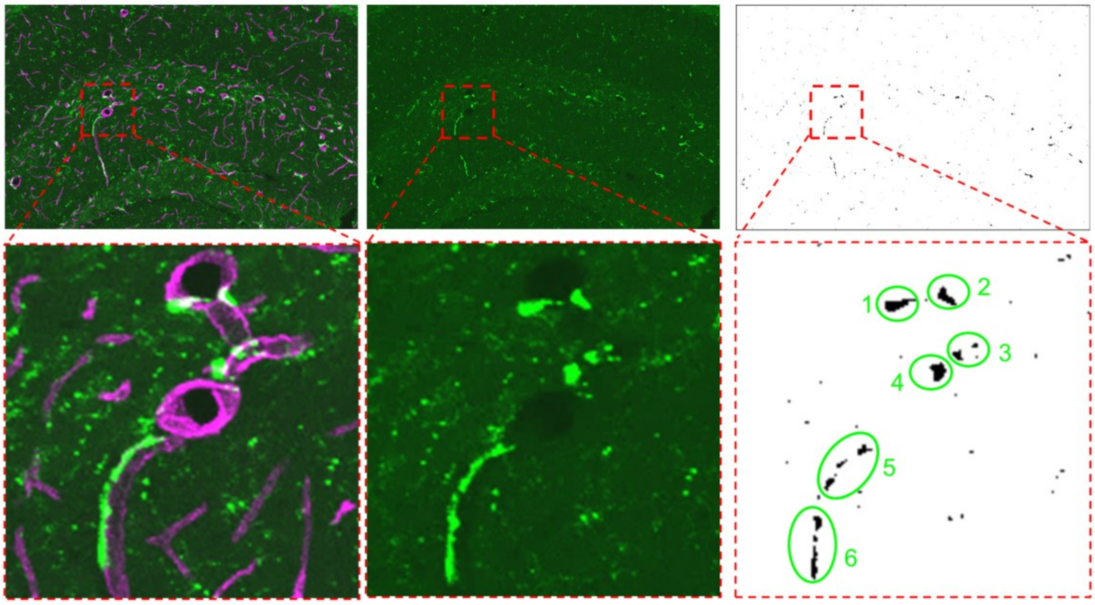


**Figure S2. Fixed-intensity threshold for outlining CD68$^{high}$ (green) cells around blood vessels (magenta, marked by PECAM-1) by Image J software.** Quantification of immunofluorescence staining was analyzed by the number of CD68$^{high}$ PVMs and fluorescent integrated intensity (IntDen) in 10× field micrographs.

**Figure S3**

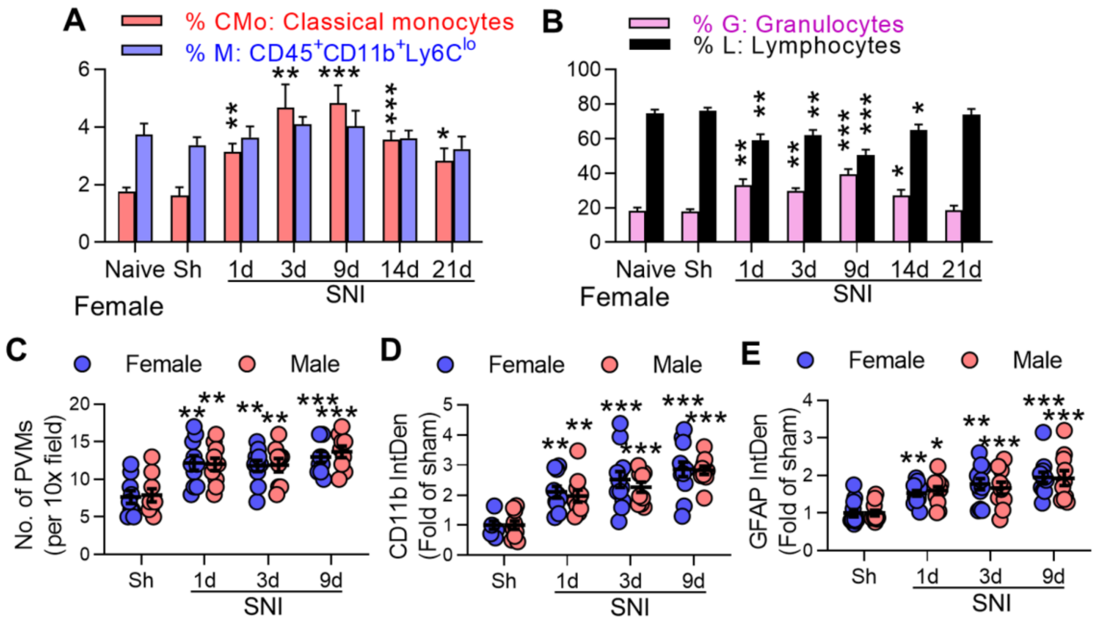


**Figure S3. The changes in blood leukocytes, hippocampal PVMs, and gliosis in female mice induced by SNI are not different from those in male mice.** (**A, B**) Time course of changes in circulating leukocyte subpopulations induced by SNI in female mice. n = 3-6 mice/group. (**C**) PVMs were increased in hippocampi of male and female SNI mice. (**D, E**) Fluorescent IntDen of CD11b (microglia marker) and GFAP astrocyte (marker) in hippocampi of male and female mice in sham and SNI mice. n = 3 mice/group, 3-4 sections/mice. *$P$ < 0.05, **$P$ < 0.01, ***$P$ < 0.001 *vs.* sham group, two-way ANOVA with Bonferroni's post hoc test.

**Figure S4**

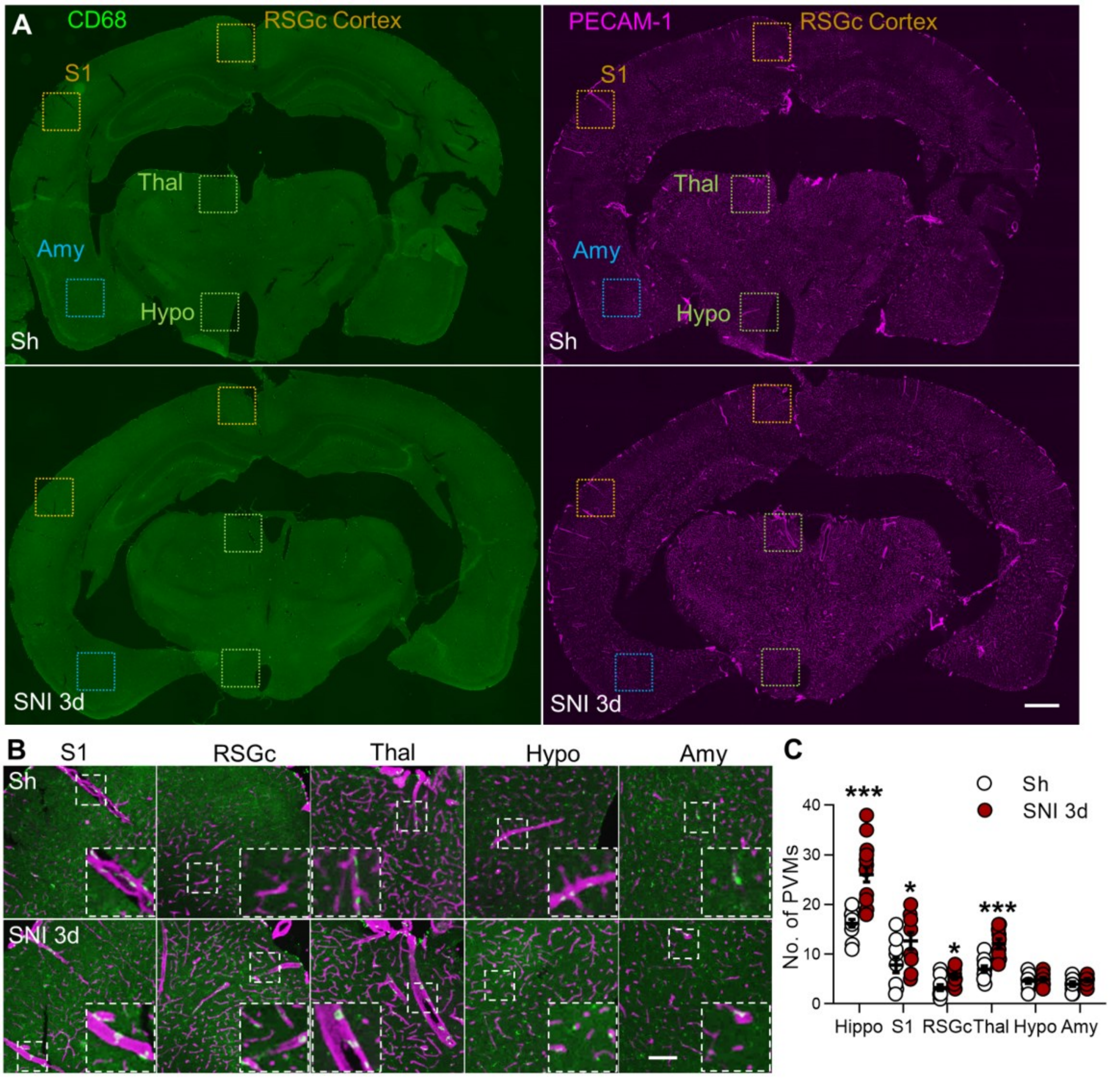


**Figure S4. SNI increases PVMs in many brain regions, but most profoundly in the hippocampus.** (**A-C**) Representative confocal images and quantitative data showing the expression of CD68 (green) and PECAM-1 (magenta) immunoreactivity in different brain regions in sham and SNI groups at 3 d after surgery (n = 3 mice/group, 3-4 images/mice). Small colored dotted boxes (in A) are magnified in B. Hippo: Hippocampus; S1: primary somatosensory cortex, RSGc: retrosplenial granular cortex, Thal: thalamus, Hypo: hypothalamus, Amy: amygdala. Scale bars, 500 µm (A), 25 µm (B). **P* < 0.05, ****P* < 0.001 vs. sham group, two-way ANOVA with Bonferroni's post hoc test.

**Figure S5**

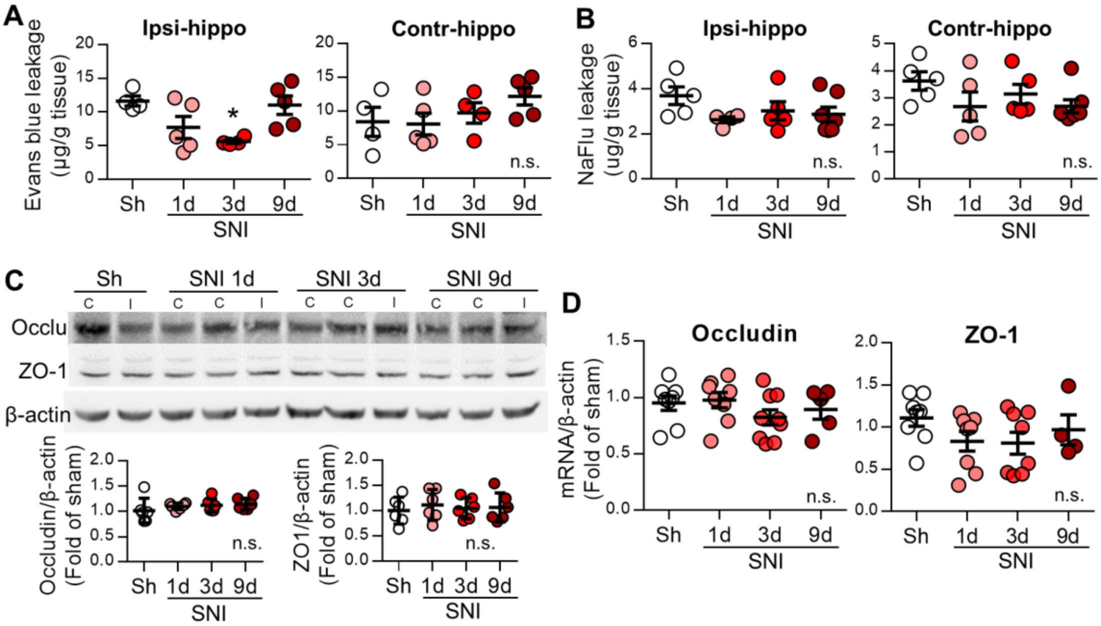


**Figure S5. Hippocampal BBB permeability was not interrupted by SNI.** (A, B) Quantification of Evans Blue (EB, A) or Fluorescein sodium (NaFlu, B) content in the bilateral hippocampi at different time points after SNI. n = 4-7 mice/group. (C) Western blot analysis for occludin-1 and ZO-1 in bilateral hippocampi. Western blot results are expressed by comparing the relative expression protein/β-actin from the sham group vs. SNI groups (n = 6 in each group). I: ipsilateral hippocampus, C: contralateral hippocampus. (D) qRT-PCR analysis for occludin-1, ZO-1 mRNA in bilateral hippocampi. n = 4-9 mice/group. Values are presented as means ± SEM. *$P < 0.05$ *vs.* sham group; n. s.: not significant; one-way ANOVA with Bonferroni's post hoc test.

**Figure S6**

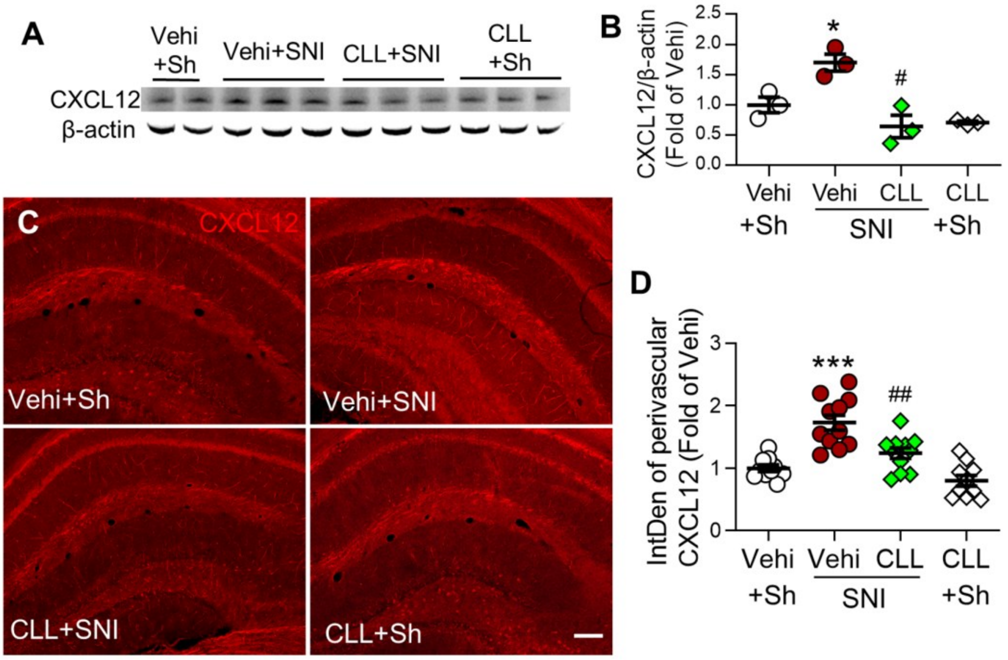


**Figure S6. The upregulation of CXCL12 in hippocampal perivascular spaces induced by SNI is prevented by deletion of circulating monocytes with clodronate.** (**A, B**) Western blots show hippocampal CXCL12 expression in various groups as indicated in sham (Sh) and SNI mice with or without clodronate at 9 days after surgery (n = 3 in each group). Vehi: vehicle. (**C, D**) Immunostaining revealed that injection of clodronate prevented the SNI-induced CXCL12 upregulation in the hippocampus but had no effect in sham mice (n = 3 mice/group, 3-4 images/mice). Scale bar, 100 µm. * $P < 0.05$, ***$P < 0.001$ *vs.* vehicle sham group, $^{\#}P < 0.05$; $^{\#\#}P < 0.01$ *vs.* SNI group, one-way ANOVA with Bonferroni's post hoc test.

**Figure S7**

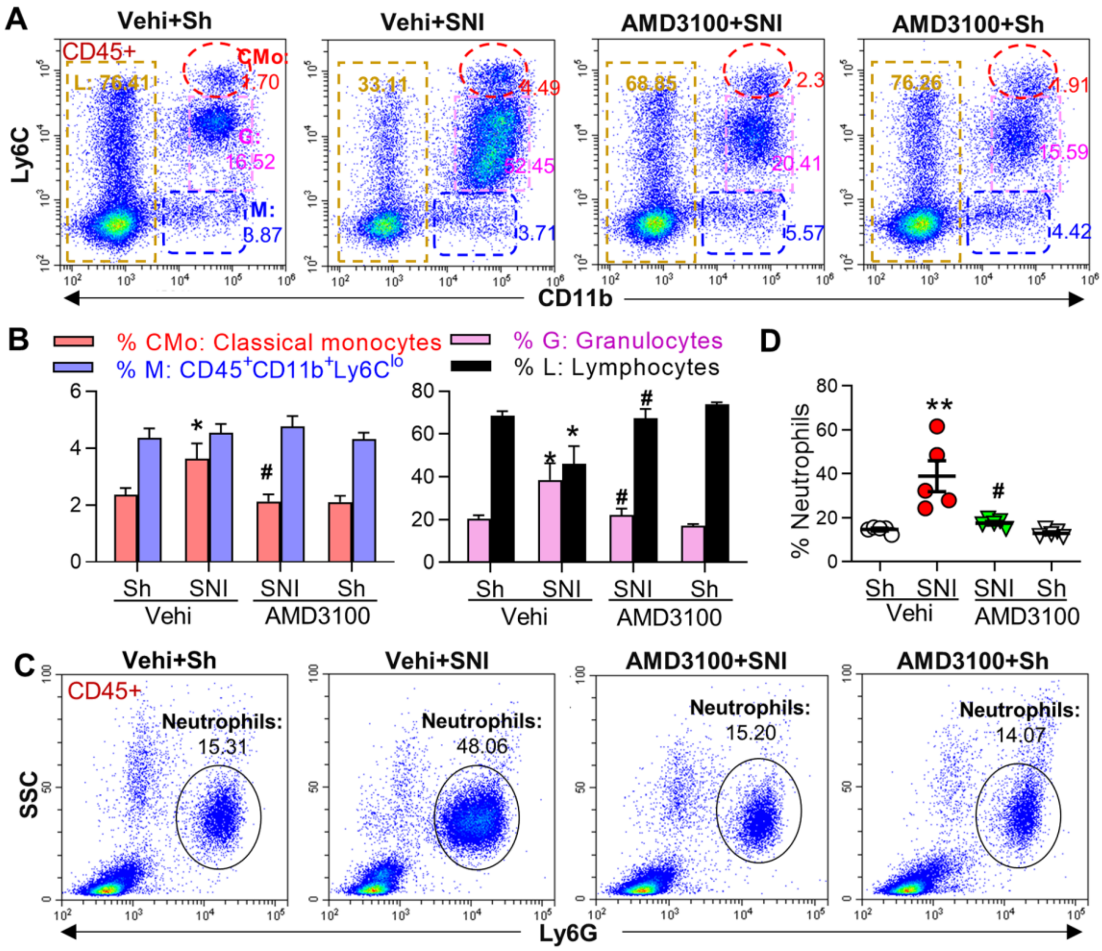


**Figure S7. Blockade of CXCR4 prevents SNI-induced changes in circulating leukocytes.** (**A-D**) Representative flow bivariate dot plots and statistical data show the changes in blood classical monocytes (CMo), $CD45^{+}CD11b^{+}L6C^{low}$ cells (M), granulocytes (G) and lymphocytes (L) and neutrophils in various groups (n = 5 mice/group). **P* < 0.05, ***P* < 0.01, *vs.* vehicle sham group, $^{\#}P < 0.05$, *vs.* vehicle SNI group, two-way (B) and one-way (D) ANOVA with Bonferroni's post hoc test.

**Table S1. Antibodies used in this study.**

| Antibody | Application (Dilution) | Catalog number | Source |
|---|---|---|---|
| rat anti-CD68 | IF (1:200) | Cat# 137006 | Biolegend, San Diego, CA, USA |
| rabbit anti-CXCL12 | IF (1:400)<br>WB (1:1000) | Cat# ab18919 | Abcam, Cambridge, UK |
| goat anti-PECAM-1 | IF (1:100) | Cat# AF3628 | R&D Systems, Minneapolis, MN, USA |
| goat anti-GFAP | IF (1:1000) | Cat# ab53554 | Abcam, Cambridge, UK |
| rat anti-CD11b | IF (1:200) | Cat# 101202 | Biolegend, San Diego, CA, USA |
| rabbit anti-P2Y12 | IF (1:400) | Cat# AS-55042A | Anaspec, Fremont, CA, USA |
| goat anti-CD13 | IF (1:200) | Cat# AF2335 | Novus, Centennial, CO, USA |
| mouse anti-β-actin | WB (1:5000) | Cat# ab170325 | Abcam, Cambridge, UK |
| mouse anti-occludin | WB (1:1000) | Cat# 331500 | Invitrogen, Carlsbad, CA, USA |
| rabbit anti-ZO-1 | WB (1:1000) | Cat# 617300 | Invitrogen, Carlsbad, CA, USA |
| AF700 rat anti-mouse CD45 | FC (1:400) | Cat# 103128 | Biolegend, San Diego, CA, USA |
| APC rat anti-mouse/human CD11b | FC (1:400) | Cat# 101212 | Biolegend, San Diego, CA, USA |
| BV510 rat anti-mouse Ly6C | FC (1:400) | Cat# 128033 | Biolegend, San Diego, CA, USA |
| BV510 rat anti-mouse Ly6G | FC (1:400)<br>IF (1:200) | Cat# 127633 | Biolegend, San Diego, CA, USA |
| APC rat anti-mouse CD3 | FC (1:400)<br>IF (1:200) | Cat# 100235 | Biolegend, San Diego, CA, USA |
| FITC hamster anti-mouse CD49b | FC (1:400)<br>IF (1:200) | Cat# 103503 | Biolegend, San Diego, CA, USA |
| AF488 donkey anti-rat IgG | IF (1:500) | Cat# A21208 | Life Technologies, Carlsbad, CA, USA |
| AF555 donkey anti-rabbit IgG | IF (1:500) | Cat# A31572 | Life Technologies, Carlsbad, CA, USA |
| AF647 donkey anti-goat IgG | IF (1:500) | Cat# A21447 | Life Technologies, Carlsbad, CA, USA |

| | | | |
|---|---|---|---|
| AF555 donkey anti-goat IgG | IF (1:500) | Cat# A21432 | Life Technologies, Carlsbad, CA, USA |
| FITC goat anti-hamster IgG | IF (1:500) | Cat# 405502 | Biolegend, San Diego, CA, USA |
| Goat anti-mouse IgG (H&L) HRP | WB (1:10000) | Cat# ab136815 | Abcam, Cambridge, UK |
| Donkey anti-rabbit IgG (H&L) HRP | WB (1:10000) | Cat# ab6802 | Abcam, Cambridge, UK |

**Table S2. The demographic data and blood test results of chronic pain patients and healthy controls.**

Note: Chronic pain patients (P), healthy controls (C), Postherpetic neuralgia (PHN), Trigeminal neuralgia (TN), Orofacial Pain (OFP), neuropathic pain (NP), chronic back pain (CBP), osteoarthritis (OA), complex regional pain syndrome (CRPS), Numeric Rating Scale (NRS), Montreal Cognitive Assessment (MoCA), leukocytes (Leu), granulocytes (G), neutrophil (Neu), lymphocyte (L), monocytes (Mo), eosinophils (Eos), basophil (Bas).

| #P | Gender | Age | Years of education | Disease diagnosis | Years of diseases | NRS | MoCA | Leu ($\times 10^9$/L) | G (%Leu) | Neu (%Leu) | L (%Leu) | Mo (%Leu) | Eos (%Leu) | Bas (%Leu) | CXCL12 (pg/mL) |
|---|---|---|---|---|---|---|---|---|---|---|---|---|---|---|---|
| 1 | M | 44 | 9 | OA | 2.00 | 5 | 25 | 4.19 | 56.10 | 52.70 | 37.70 | 6.20 | 2.40 | 1.00 | 58.33 |
| 2 | F | 81 | 9 | CRPS | 5.00 | 6 | 24 | 5.04 | 77.10 | 74.90 | 17.10 | 5.80 | 1.80 | 0.40 | 153.75 |
| 3 | M | 38 | 12 | CBP | 1.00 | 7 | 23 | 7.68 | 60.30 | 54.00 | 31.00 | 8.70 | 4.90 | 1.40 | 175.42 |
| 4 | F | 54 | 7 | OA | 2.00 | 6 | 23 | 4.25 | 53.90 | 51.10 | 37.60 | 8.50 | 2.10 | 0.70 | 177.92 |
| 5 | M | 80 | 9 | PHN | 0.25 | 9 | 22 | 6.43 | 71.40 | 69.10 | 22.70 | 5.90 | 2.00 | 0.30 | 179.17 |
| 6 | F | 75 | 12 | PHN, OA | 1.00 | 4 | 23 | 9.83 | 74.90 | 74.80 | 18.10 | 7.00 | 0.00 | 0.10 | 137.92 |
| 7 | M | 59 | 12 | PHN | 10.00 | 8 | 22 | 11.88 | 76.40 | 72.20 | 13.00 | 10.60 | 3.60 | 0.60 | 373.33 |
| 8 | F | 70 | 9 | OA, CRPS | 0.50 | 5 | 23 | 4.12 | 64.80 | 63.10 | 27.40 | 7.80 | 1.50 | 0.20 | 434.17 |
| 9 | F | 25 | 16 | CBP | 0.25 | 5 | 26 | 11.26 | 68.80 | 67.80 | 22.60 | 8.60 | 0.30 | 0.70 | 190.00 |
| 10 | M | 37 | 12 | NP | 6.00 | 6 | 26 | 6.63 | 65.90 | 64.40 | 27.80 | 6.30 | 0.90 | 0.60 | 87.92 |
| 11 | F | 51 | 6 | CBP | 20.00 | 4 | 22 | 5.42 | 55.60 | 53.90 | 38.70 | 5.70 | 1.30 | 0.40 | 145.83 |
| 12 | M | 49 | 7 | CRPS | 0.42 | 7 | 20 | 3.65 | 58.20 | 54.10 | 31.80 | 10.00 | 3.40 | 0.70 | 185.83 |
| 13 | M | 45 | 9 | OFP, CRPS | 0.50 | 6 | 22 | 5.92 | 76.80 | 76.00 | 18.60 | 4.60 | 0.30 | 0.50 | 586.25 |
| 14 | M | 34 | 16 | NP | 0.25 | 6 | 29 | 5.92 | 61.60 | 59.90 | 32.10 | 6.30 | 1.00 | 0.70 | 157.08 |
| 15 | M | 63 | 16 | CBP, CRPS | 0.25 | 6 | 25 | 5.17 | 67.00 | 64.10 | 25.10 | 7.90 | 2.50 | 0.40 | 426.67 |
| 16 | F | 58 | 6 | PHN, CRPS | 10.00 | 7 | 20 | 7.14 | 61.60 | 61.20 | 30.00 | 8.40 | 0.10 | 0.30 | 766.67 |
| 17 | F | 19 | 13 | CRPS | 1.00 | 7 | 30 | 7.88 | 62.30 | 59.70 | 26.00 | 11.70 | 2.20 | 0.40 | 125.00 |
| 18 | F | 64 | 12 | NP | 0.25 | 5 | 24 | 5.79 | 59.60 | 57.40 | 34.70 | 5.70 | 1.60 | 0.60 | 139.58 |
| 19 | F | 52 | 12 | OA, CRPS | 0.50 | 6 | 26 | 4.95 | 65.30 | 63.30 | 23.80 | 10.90 | 1.60 | 0.40 | 805.42 |
| 20 | M | 67 | 9 | OA | 1.00 | 5 | 22 | 5.96 | 70.40 | 66.90 | 22.80 | 6.80 | 2.90 | 0.60 | 19.17 |
| 21 | F | 67 | 9 | CBP | 0.25 | 5 | 25 | 9.01 | 65.50 | 63.60 | 28.60 | 5.90 | 1.60 | 0.30 | 104.58 |
| 22 | F | 55 | 7 | CBP | 0.50 | 5 | 21 | 8.17 | 73.00 | 67.50 | 21.40 | 5.60 | 4.00 | 1.50 | 104.58 |
| 23 | F | 63 | 6 | OA | 1.00 | 5 | 22 | 6.38 | 66.50 | 59.50 | 26.50 | 7.00 | 6.60 | 0.40 | 102.08 |
| 24 | F | 49 | 6 | TN | 2.00 | 5 | 23 | 7.28 | 76.00 | 74.50 | 16.30 | 7.70 | 1.20 | 0.30 | 85.83 |

| | | | | | | | | | | | | | | | |
|---|---|---|---|---|---|---|---|---|---|---|---|---|---|---|---|
| 25 | M | 83 | 6 | CRPS | 40.00 | 9 | 21 | 6.96 | 71.60 | 70.60 | 22.10 | 6.30 | 0.90 | 0.10 | 150.42 |
| 26 | F | 39 | 12 | CBP | 0.25 | 8 | 27 | 12.67 | 77.90 | 71.90 | 17.40 | 4.70 | 3.70 | 2.30 | 114.58 |
| 27 | M | 76 | 6 | OA, CBP | 2.00 | 5 | 22 | 5.33 | 58.40 | 53.50 | 33.20 | 8.40 | 4.50 | 0.40 | 141.67 |
| 28 | M | 52 | 9 | CBP | 1.00 | 6 | 28 | 7.48 | 68.40 | 66.70 | 25.20 | 6.40 | 1.60 | 0.10 | 170.83 |
| 29 | F | 42 | 6 | OA, CBP | 0.42 | 7 | 22 | 6.52 | 66.30 | 64.60 | 26.20 | 7.50 | 1.40 | 0.30 | 116.67 |
| 30 | M | 28 | 16 | NP, CBP | 1.00 | 5 | 26 | 6.01 | 65.00 | 63.80 | 28.00 | 7.00 | 1.20 | 0.00 | 186.67 |

| #C | Gender | Age | Years of education | Disease diagnosis | Years of diseases | NRS | MoCA | Leu $(\times 10^9/L)$ | G (%Leu) | Neu (%Leu) | L (%Leu) | Mo (%Leu) | Eos (%Leu) | Bas (%Leu) | CXCL12 (pg/mL) |
|---|---|---|---|---|---|---|---|---|---|---|---|---|---|---|---|
| 1 | M | 56 | 9 | - | - | 0 | 28 | 5.81 | 60.50 | 57.40 | 33.60 | 5.90 | 2.40 | 0.70 | 31.74 |
| 2 | F | 50 | 9 | - | - | 0 | 28 | 4.74 | 59.50 | 57.20 | 34.30 | 6.20 | 1.30 | 1.00 | 8.01 |
| 3 | F | 42 | 12 | - | - | 0 | 29 | 5.34 | 58.90 | 55.40 | 34.60 | 6.50 | 2.80 | 0.70 | 11.89 |
| 4 | F | 47 | 7 | - | - | 0 | 26 | 5.11 | 52.90 | 49.50 | 39.20 | 7.90 | 2.80 | 0.60 | 17.85 |
| 5 | F | 49 | 12 | - | - | 0 | 26 | 7.69 | 63.50 | 61.20 | 32.40 | 4.10 | 1.80 | 0.50 | 31.80 |
| 6 | M | 42 | 9 | - | - | 0 | 28 | 5.38 | 53.10 | 45.70 | 41.30 | 5.60 | 6.70 | 0.70 | 23.87 |
| 7 | F | 35 | 16 | - | - | 0 | 28 | 4.85 | 55.70 | 54.50 | 39.60 | 4.70 | 0.80 | 0.40 | 21.97 |
| 8 | M | 44 | 12 | - | - | 0 | 28 | 7.76 | 53.20 | 50.90 | 39.60 | 7.20 | 1.60 | 0.70 | 23.70 |
| 9 | M | 59 | 9 | - | - | 0 | 29 | 4.75 | 60.10 | 55.00 | 34.10 | 5.80 | 4.40 | 0.70 | 16.96 |
| 10 | F | 50 | 9 | - | - | 0 | 27 | 9.36 | 70.10 | 68.00 | 25.00 | 4.90 | 1.50 | 0.60 | 20.68 |
| 11 | F | 57 | 12 | - | - | 0 | 29 | 6.12 | 55.20 | 52.20 | 39.00 | 5.80 | 2.60 | 0.40 | 38.57 |
| 12 | F | 52 | 9 | - | - | 0 | 30 | 5.16 | 50.60 | 48.70 | 44.30 | 5.10 | 1.50 | 0.40 | 31.67 |
| 13 | F | 66 | 9 | - | - | 0 | 27 | 5.60 | 62.10 | 57.20 | 30.10 | 7.80 | 4.30 | 0.60 | 40.00 |
| 14 | F | 37 | 9 | - | - | 0 | 27 | 5.15 | 64.20 | 63.10 | 29.60 | 6.20 | 0.90 | 0.20 | 15.00 |
| 15 | M | 45 | 12 | - | - | 0 | 26 | 5.39 | 47.20 | 44.00 | 45.20 | 7.60 | 2.20 | 1.00 | 0.83 |
| 16 | F | 57 | 12 | - | - | 0 | 26 | 6.30 | 49.80 | 48.50 | 42.40 | 7.80 | 1.30 | 0.00 | 35.00 |
| 17 | F | 60 | 9 | - | - | 0 | 29 | 6.53 | 55.20 | 53.20 | 39.70 | 5.10 | 2.00 | 0.00 | 40.00 |
| 18 | M | 57 | 6 | - | - | 0 | 27 | 4.58 | 70.50 | 67.50 | 23.90 | 5.60 | 2.40 | 0.60 | 29.89 |
| 19 | F | 43 | 6 | - | - | 0 | 26 | 6.54 | 63.90 | 60.40 | 29.60 | 6.50 | 3.10 | 0.40 | 30.69 |
| 20 | F | 58 | 6 | - | - | 0 | 28 | 5.22 | 60.30 | 58.80 | 33.40 | 6.30 | 0.90 | 0.60 | 40.83 |
| 21 | M | 40 | 12 | - | - | 0 | 30 | 6.17 | 59.30 | 57.30 | 34.00 | 6.70 | 1.40 | 0.60 | 78.72 |
| 22 | M | 38 | 7 | - | - | 0 | 30 | 6.27 | 61.30 | 58.20 | 32.60 | 6.10 | 2.70 | 0.40 | 10.03 |
| 23 | M | 47 | 6 | - | - | 0 | 25 | 5.70 | 57.40 | 55.50 | 34.60 | 8.00 | 1.40 | 0.50 | 58.04 |
| 24 | M | 54 | 16 | - | - | 0 | 29 | 7.25 | 58.90 | 56.80 | 34.90 | 6.20 | 1.60 | 0.50 | 39.59 |
| 25 | M | 44 | 12 | - | - | 0 | 29 | 6.84 | 57.40 | 54.60 | 36.20 | 6.40 | 1.90 | 0.90 | 32.35 |
| 26 | F | 44 | 9 | - | - | 0 | 26 | 3.96 | 50.50 | 46.40 | 42.50 | 7.00 | 3.30 | 0.80 | 28.00 |
| 27 | F | 36 | 12 | - | - | 0 | 28 | 4.39 | 55.10 | 53.50 | 38.90 | 6.00 | 1.00 | 0.60 | 32.20 |
| 28 | F | 46 | 9 | - | - | 0 | 27 | 4.52 | 55.50 | 52.30 | 36.80 | 7.70 | 2.60 | 0.60 | 49.99 |
| 29 | F | 53 | 9 | - | - | 0 | 26 | 8.19 | 72.50 | 71.20 | 22.20 | 5.30 | 1.20 | 0.10 | 2.50 |
| 30 | M | 53 | 7 | - | - | 0 | 29 | 8.27 | 63.00 | 49.20 | 30.60 | 6.40 | 13.30 | 0.50 | 28.33 |
| 31 | F | 51 | 12 | - | - | 0 | 28 | 5.57 | 58.60 | 55.40 | 35.50 | 5.90 | 3.20 | 0.00 | 16.67 |
| 32 | F | 47 | 6 | - | - | 0 | 27 | 6.75 | 64.00 | 63.00 | 28.30 | 7.70 | 0.90 | 0.10 | 19.17 |
| 33 | M | 52 | 12 | - | - | 0 | 28 | 11.15 | 60.60 | 59.30 | 33.40 | 6.00 | 1.20 | 0.10 | 0.00 |
| 34 | M | 73 | 8 | - | - | 0 | 27 | 10.25 | 78.40 | 71.90 | 16.00 | 5.60 | 6.40 | 0.10 | 51.67 |
| 35 | F | 37 | 9 | - | - | 0 | 29 | 6.44 | 57.20 | 56.20 | 36.60 | 6.20 | 0.80 | 0.20 | 40.00 |

| | | | | | | | | | | | | | | | |
|---|---|---|---|---|---|---|---|---|---|---|---|---|---|---|---|
| 36 | F | 29 | 16 | - | - | 0 | 30 | 4.34 | 67.40 | 66.00 | 29.60 | 3.00 | 1.10 | 0.30 | 56.67 |
| 37 | M | 71 | 12 | - | - | 0 | 28 | 7.53 | 87.90 | 86.60 | 7.60 | 4.50 | 0.90 | 0.40 | 39.80 |
| 38 | F | 27 | 9 | - | - | 0 | 26 | 5.99 | 50.20 | 48.70 | 42.20 | 7.60 | 1.00 | 0.50 | 16.18 |
| 39 | M | 44 | 9 | - | - | 0 | 28 | 7.23 | 62.30 | 58.50 | 31.60 | 6.10 | 2.90 | 0.90 | 14.00 |
| 40 | F | 58 | 12 | - | - | 0 | 27 | 6.08 | 51.00 | 48.40 | 43.00 | 6.00 | 1.90 | 0.70 | 52.88 |
